# Lipid‐Driven OLR1/FOXM1/FGF19 Axis Orchestrates Crosstalk in an Epithelial‐Fibroblast Positive Feedback Promoting Progesterone Resistance in Endometrial Cancer

**DOI:** 10.1002/advs.202511943

**Published:** 2025-11-21

**Authors:** Xingchen Li, Yue Qi, Yuman Wu, Xinyi Bi, Yiqin Wang, Jiaqi Wang, Jingyuan Wang, Lingpu Zhang, Haihua Xiao, Jianliu Wang

**Affiliations:** ^1^ Department of Obstetrics and Gynecology Peking University People's Hospital Beijing 100044 China; ^2^ Beijing National Laboratory for Molecular Sciences State Key Laboratory of Polymer Physics and Chemistry Institute of Chemistry Chinese Academy of Sciences Beijing 100190 China

**Keywords:** endometrial cancer, fertility preservation, OLR1, oxLDL, progesterone resistance

## Abstract

Progesterone resistance (ProR) remains a major obstacle in the conservative management of endometrial cancer (EC). Here, a metabolic‐stromal signaling loop centered on the OLR1/FOXM1/FGF19 axis is identified that drives progesterone resistance in EC. Single‐cell transcriptomic profiling first revealed a striking correlation between epithelial cells and fibroblasts in EC tissues with ProR. Tumor epithelial cells display profound alterations in lipid metabolism, whereas fibroblasts exhibited enhanced oxidative stress signatures. Clinical samples analyses indicated that oxidized low density lipoprotein (oxLDL), a product of LDL oxidation, is associated with adverse outcomes. The binding of oxLDL to its receptor OLR1 promoted the expression of FOXM1, a transcription factor that directly upregulates fibroblast growth factor 19 (FGF19). Immunofluorescence confirmed not only the spatial co‐localization of epithelial cells and fibroblasts but also the enrichment of OLR1 within epithelial compartments. Furthermore, treatment with the antioxidant resveratrol (RSV) and its nanoformulation (RSV‐NPs) markedly inhibited tumor growth in mice with lipid metabolic disorders, highlighting their potential to counteract progesterone resistance by disrupting this OLR1/FOXM1/FGF19 axis. This work highlights the therapeutic potential of targeting the tumor–stroma metabolic axis to increase progesterone sensitivity and improve outcomes in EC patients with fertility‐preserving demands.

## Graphical abstract

It is revealed that a metabolic–stromal loop driven by oxLDL–OLR1–FOXM1–FGF19 signaling promotes progesterone resistance in endometrial cancer. OxLDL activates epithelial–fibroblast crosstalk, enhancing proliferation and hormonal insensitivity. Disruption of this circuit, especially with resveratrol, restores MPA sensitivity, highlighting a potential strategy to overcome hormone resistance via targeting tumor–fibroblast metabolic interactions.

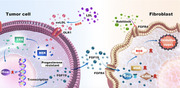

## 1 Introduction

Endometrial cancer (EC) ranks among the most prevalent gynecologic malignancies, with rising incidence rates, particularly among younger women.^[^ ^1^ ^]^ While hysterectomy remains the standard treatment, it results in irreversible fertility loss, a critical concern for premenopausal patients desiring future childbearing.^[^ ^2^ ^]^ High‐dose progestin therapy is widely employed for fertility preservation, notably medroxyprogesterone acetate (MPA), demonstrating favorable response rates in early‐stage disease. However, nearly 30% of patients exhibit primary progestin resistance, and another subset develops acquired resistance during treatment, leading to disease progression and necessitating definitive surgery.^[^ ^3^ ^]^ The underlying mechanisms of resistance remain poorly understood, and current hypotheses involve progesterone receptor dysregulation and aberrant signaling pathways.^[^ ^4^ ^]^ Given the clinical urgency to improve treatment outcomes, elucidating the molecular drivers of progesterone resistance (ProR) is essential to enhance therapeutic sensitivity and preserve fertility in young EC patients.

Recent studies have shown that lipid metabolism plays a key role in tumor progression and therapy resistance.^[^ ^5^ ^]^ EC is often linked to metabolic disorders, and showing altered lipid metabolism that contributes to hormonal therapy resistance.^[^ ^6^ ^]^ Oxidized low‐density lipoprotein (oxLDL) accumulates in tumors, activating cancer‐promoting pathways like MAPK.^[^ ^7^ ^]^ The oxLDL receptor OLR1 (also named LOX‐1) drives cancer progression in various cancers. For example, oxLDL/LOX‐1 signaling drives prostate cancer progression by inducing epithelial‐mesenchymal transition(EMT) and enhancing tumor invasion, providing a mechanistic link between obesity‐related lipid metabolism and aggressive prostate cancer.^[^ ^8^ ^]^ Similarly, OLR1 overexpression in breast cancer correlates with poor prognosis and immune evasion by promoting M2 macrophage polarization, suggesting its potential as a therapeutic target to counteract tumor‐associated immunosuppression.^[^ ^9^ ^]^ However, how OLR1 affects ProR in EC remains unknown, leaving a gap in fertility‐preserving treatments.

Tumor cells reprogram lipid metabolism to support their growth and shape the tumor microenvironment. Cancer‐related lipids trigger excessive fibroblast growth factor(FGF) release, activating nearby fibroblasts.^[^ ^10^ ^]^ FGF signaling causes oxidative stress in fibroblasts, leading to reactive oxygen species (ROS) buildup. This stress triggers two key responses: the p53 pathway increases Bcl‐2 to block cell death, while MAPK/ERK signaling boosts inflammation and ECM breakdown via IL‐6 and MMP‐9.^[^ ^11^, ^12^ ^]^ Tumor FGFs keep fibroblasts active, while fibroblast signals like TGF‐β and oxLDL make tumors more aggressive and therapy‐resistant.^[^ ^13^ ^]^ In EC, this lipid‐FGF‐ROS axis may weaken progesterone treatment by creating a protective tumor environment. Breaking this tumor‐stroma link could therefore help overcome hormonal resistance in EC with faulty lipid metabolism.

This study leverages single‐cell RNA sequencing data from fertility‐preserving EC patients to elucidate the mechanism by which oxLDL/OLR1 signaling drives ProR. We identified a critical metabolic‐stromal crosstalk between epithelial cells and fibroblasts in resistant tissues, demonstrating that OLR1 in tumor epithelial cells not only enhances intrinsic ProR, but also upregulates FGF19 transcription and secretion. Secreted FGF19 activates stromal fibroblasts, inducing oxidative stress that further converts LDL to oxLDL, thereby establishing a feedforward loop that perpetuates hormonal resistance. Importantly, we revealed that combined treatment with resveratrol (RSV) and lipid‐lowering statins effectively disrupts this vicious cycle by simultaneously targeting OLR1‐mediated lipid uptake and fibroblast‐derived oxidative stress. Our findings provide a clinically actionable strategy to restore treatment sensitivity in EC.

## 2 Results

### 2.1 Single‐Cell Transcriptomic Profiling of EC Reveals Cellular Heterogeneity Between Drug‐Resistant and Sensitive Tumors

**Figure 1:**
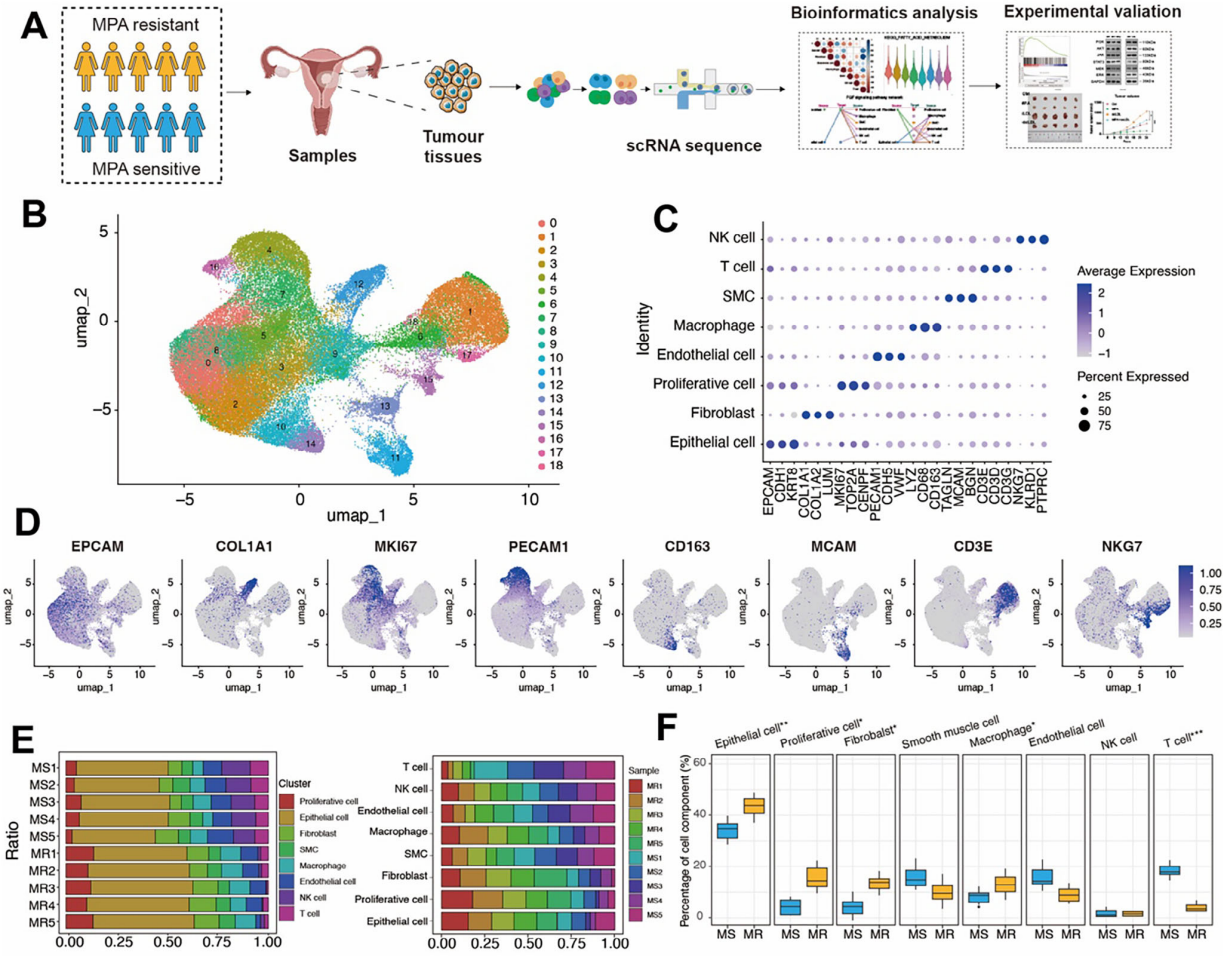
The transcriptomic landscape at the resolution of scRNA‐seq from EC patients with different MPA sensitivity. A) The schematic diagram illustrates the research design in this study. A total of 10 EC surgical tissues were collected for single‐cell RNA sequencing, including 5 MS EC tissues and 5 MR tissues. B) Uniform manifold approximation and projection (UMAP) plots displayed 19 identified major cell types from 108557 single cells. C) Dot plots showed the normalized expression levels of marker genes in each cell subtype. D) Expression levels of selected known marker genes across 108557 unsorted cells are illustrated in UMAP plots from both sensitive and resistant tissue in EC patients. E) The proportion of each annotated cell subtype in different individuals and the proportion of each individual in different cell clusters. F) The comparison of the proportion of each annotated cell cluster across different MPA sensitivity (*n* = 5 for each group). P‐values were determined using Student's t‐test. MS, MPA sensitive, MR, MPA resistance. ^*^ *p* < 0.05, ^**^ *p* < 0.01, ^***^ *p* < 0.001.

To comprehensively characterize the tumor microenvironment (TME) of EC with differential treatment responses, we performed single‐cell RNA sequencing (scRNA‐seq) on paired tissue samples from five MPA‐resistant (MR) and five MPA‐sensitive (MS) patients (**Figure** **1**A). Following rigorous quality control, we analyzed 108 557 high‐quality cells (resistant group: 56 475; sensitive group: 52 082) using UMAP dimensionality reduction, which revealed 19 major cell populations (Figure 1B). The cells were classified into eight major cell types (Figure 1C), including epithelial cells identified by the expression of EPCAM, CDH1, KRT8; fibroblasts identified by the expression of COL1A1, COL1A2, LUM; proliferative cells marked by MKI67, TOP2A, CENPF; endothelial cells (PECAM1, CDH5, VWF); macrophages (LYZ, CD68, CD163); smooth muscle cells (TAGLN, MCAM, BGN); T cells (CD3D, CD3E, CD3G); and NK cells (NKG7, KLRD1, PTPRC).^[^ ^14^, ^15^, ^16^, ^17^ ^]^ Marker gene analysis demonstrated distinct expression patterns across clusters (Figure 1D; Figure S1, Supporting Information). It is well established that such proliferative clusters emerge computationally due to the strong signal from actively cycling cells across various lineages, rather than representing a unique cellular origin. Comparative analysis revealed significant compositional differences between resistant and sensitive groups. The resistant TME showed increased proportions of immunosuppressive cell types (Epithelial cell: 42.5% vs 35.6%, *p*<0.01; Proliferative cells: 14.3% vs 5.6%, *p *= 0.02; fibroblasts (14.5% vs 4.3%, *p* = 0.03), and macrophage: 12.4% vs 8.4%, *p* = 0.04) whereas sensitive tumors contained more T cells (18.7% vs 4.3%, *p* = 0.04, Figure 1E,F). This dichotomous pattern suggests that ProR arises through coordinated epithelial‐stromal activation and immune evasion, whereas treatment sensitivity correlates with preserved anti‐tumor immunity.

### 2.2 Fibroblast‐Epithelial Interaction Correlates with Poor Clinical Outcomes

**Figure 2:**
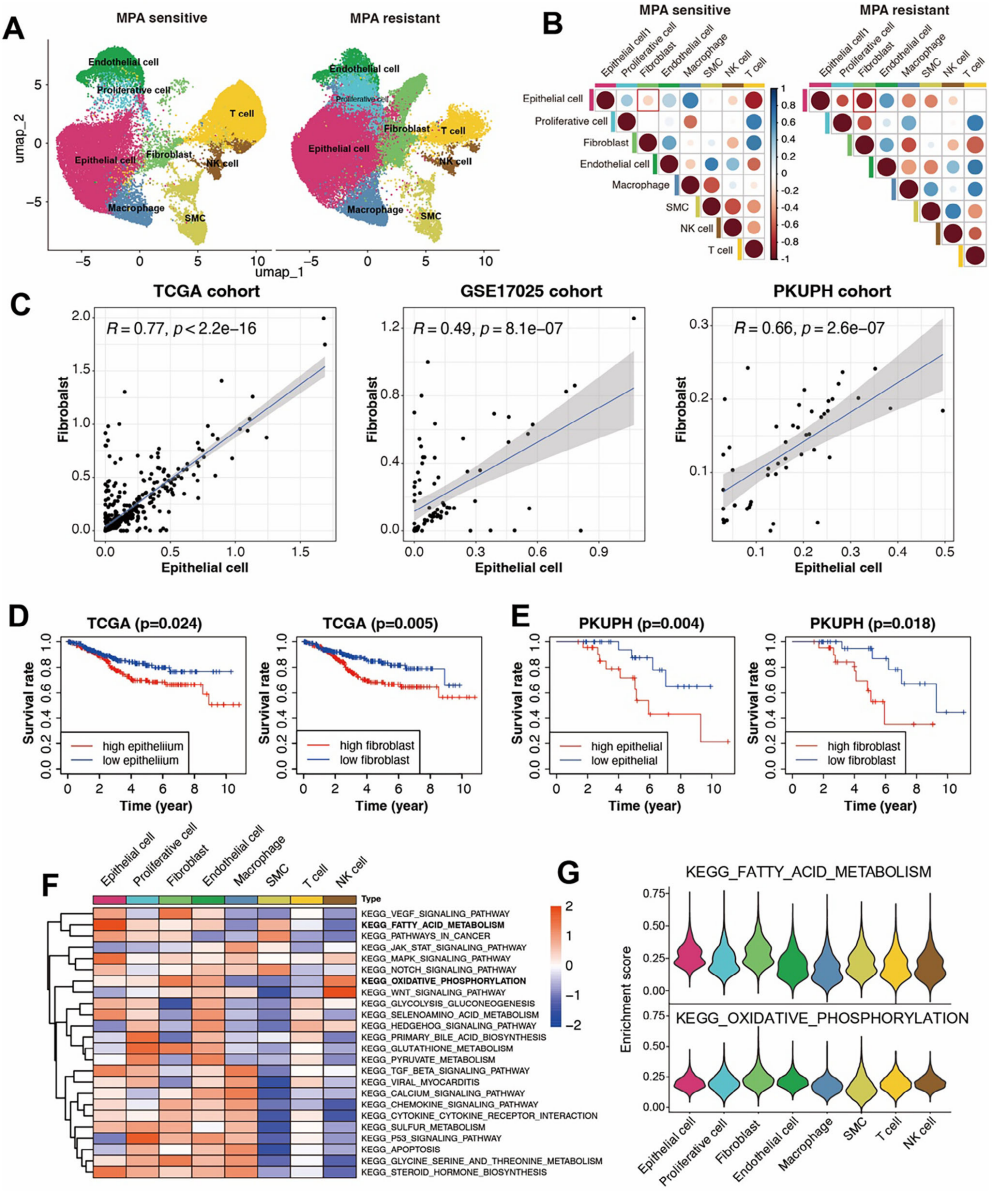
Correlation and function between epithelial cells and fibroblasts in tumor infiltration associated with the different MPA sensitivity. A) Split UMAP plots for detailed clusters showing MR and MS patients. B) The proportion of EC cohorts with positive (Spearman correlation; correlation coefficient in red) or negative (in blue) correlation for the infiltration of pairwise cell types subgroups with different MPA sensitivity. C) Scatter plots show the correlation between the infiltration of epithelial cells and fibroblast cells across 3 independent datasets with RNA‐seq of EC, including TCGA‐UCEC (*n* = 532); GSE17025 (*n* = 91); and PKUPH (*n* = 49). The oblique line and R represent the average correlation coefficient. D, E) The Kaplan–Meier curves showed that patients with higher infiltration of epithelial cells (left) and fibroblast cells (right) are associated with worse OS in D) TCGA cohort and E) PKUPH cohort, adjusted by age and tumor stage, statistically tested using the log‐rank test. F) The heatmap illustrates the enrichment of signaling pathways in different cell subtypes, analyzed by GSVA. G) Enrichment score of fatty acid metabolism and oxidative phosphorylation pathways of each cell type in the scRNA‐seq data. A two‐sided log‐rank test *p* < 0.05 is considered a statistically significant difference in Kaplan–Meier survival analysis. MS, MPA sensitive, MR, MPA resistance.

To delineate cellular heterogeneity between MR and MS EC, we performed UMAP analysis, split by MPA sensitivity, revealing distinct clustering patterns (**Figure** **2**A). Functional enrichment analysis(including GO and KEGG) of differentially expressed genes (DEGs) between MR and MS epithelial cells from scRNA sequencing, revealed significant enrichment in biological processes, including fatty acid metabolic process, lipase activity, and chemokine signaling pathway (Figure S2A,B, Supporting Information). Among the eight identified cell types, epithelial cells and fibroblasts exhibited the strongest correlation in the MR group (Figure 2B), suggesting a potential epithelial‐stromal crosstalk in the resistant group. To validate these findings, we employed CIBERSORT to deconvolute bulk RNA‐seq data from TCGA‐UCEC, GSE17025, and PKUPH cohorts. Pearson correlation analysis confirmed a robust epithelial‐fibroblast association across independent datasets (Figure 2C). To further verify the relationship between the identified cell types and survival, we used the CIBERSORT algorithm to calculate the proportion of each cell type for each patient in the bulk RNA data of the TCGA database. The survival differences between the two groups with high and low cell proportions were compared. Clinically, high fibroblast and epithelial cell abundance predicted worse overall survival in both TCGA (*p* = 0.024 in epithelial cell s, and *p* = 0.005 for fibroblasts, Figure 2D) and PKUPH cohorts (*p* = 0.044 in epithelial cells, and *p* = 0.018 for fibroblasts, Figure 2E), underscoring their prognostic relevance. Through differential genes analysis of the sequencing results of two groups of scRNA data, KEGG pathway analysis of cell type‐specific gene signatures revealed that epithelial cell‐enriched genes were associated with fatty acid metabolism, which was the most significant pathway in the KEGG analysis. Meanwhile, fibroblast‐specific genes were linked to oxidative phosphorylation (Figure 2F). Violin plots further validated these enrichment patterns (Figure 2G). Furthermore, subclustering specifically on epithelial cells was performed. This analysis demonstrated that within the MPA‐resistant epithelial cells, the expression of lipid metabolism‐related genes, including ACSL1, APOE, ALDH1A1, and ALOX5, were significantly upregulated (Figure S2C, Supporting Information). These results illustrated that metabolic reprogramming (epithelial‐driven) and oxidative stress (fibroblast‐driven) jointly contributed to aggressive disease phenotypes, but the mechanism is still ambiguous.

### 2.3 FGF Signaling from Epithelial Cells Regulates Oxidative Stress in Fibroblasts

**Figure 3:**
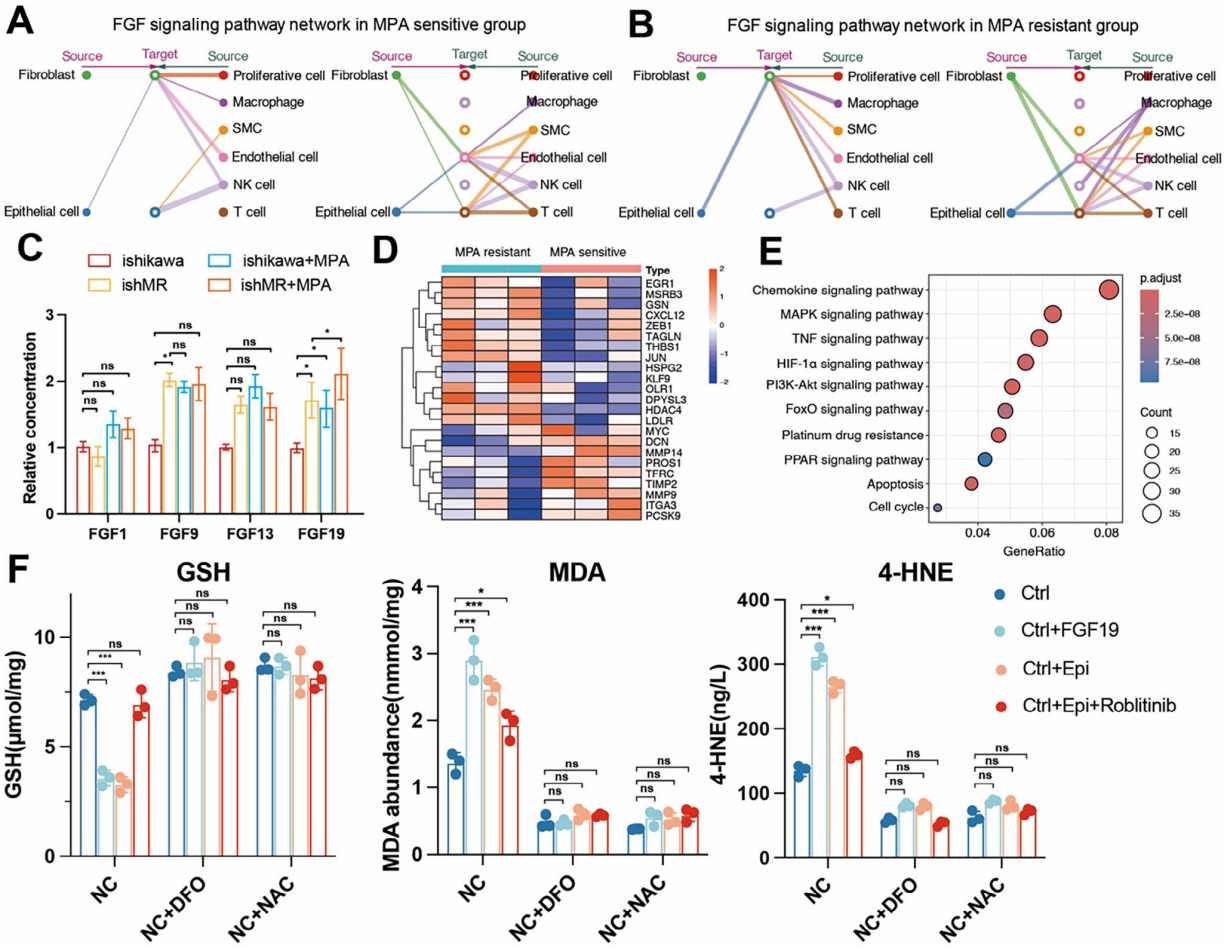
Functional differences and validation between epithelial cells and fibroblasts for FGF signaling pathways. A, B) Plots illustrate the differential number of interactions between any two cell types in the MS and resistant subgroups. The thickness of the lines represents the strength of interactions. C) ELISA analyses of common FGF level in Ishikawa cell lines with different MPA sensitivity (*n* = 3 for each group). D) Lipid‐metabolism related DEGs between MS and resistant epithelial cells. E) KEGG analysis with DEGs between MS and resistant groups in epithelial cells from scRNA data. F) Ishikawa was treated with FGF19, co‐cultured with epithelial cells, or added with roblitinib (1 µm) in the absence or presence of DFO (100 µm) or NAC (1 mm) for 24 h. Then intracellular GSH (left panel), MDA (middle panel), or 4‐HNE (right panel) were assayed in the four groups. All histogram chart data are presented as the mean ± SD. Statistical analyses were performed by Graphpad Prism 10.2.3 using the two‐tailed Student's t‐test to detect differences between the groups. *n* = 3 biological replicates. MR, MPA resistant, MS, MPA sensitive. ishMR, MR Ishikawa. ns, not significant, ^*^ *p* < 0.05, ^**^ *p* < 0.01, ^***^ *p* < 0.001.

In order to further investigate the intercellular communication between fibroblasts and epithelial cells in EC, we used the CellChat package to analyze the intercellular communication between two groups of samples with different sensitivity to MPA. CellChat analysis of the eight major cell types revealed that epithelial cells and fibroblasts exhibited a higher number of interactions in the MR group, indicating robust bidirectional communication (Figure S3A, Supporting Information). Pathway enrichment identified the FGF signaling network as the dominant mediator of this crosstalk, with epithelial cells serving as the primary signal source in the MR group compared to the MS group (**Figure** **3**A,B). Given the established role of FGF signaling in fibroblast activation and therapy resistance,^[^ ^18^ ^]^ we investigated its association with progesterone receptor (PR) status, a key determinant of hormonal therapy response.^[^ ^19^ ^]^ In RNA level of both TCGA and PKUPH cohorts, differential expression analysis of FGF ligands in PR‐high vs PR‐low tumors identified FGF1, FGF9, FGF13, and FGF19 as significantly upregulated in PR‐low (hormone‐resistant) cases (*p*‐value < 0.05, Figure S3B, Supporting Information), indicating that these FGFs may play an essential role in MR. In vitro validation using EC‐fibroblast co‐cultures confirmed that FGF19 secretion was progressively elevated in resistant models (*p*‐value < 0.05, Figure 3C). Functional assays demonstrated that both conditioned medium from resistant epithelial cells and FGF19 exhibited a chemotactic effect on fibroblasts (Figure S3C, Supporting Information). It has been reported that FGFR4 serves as the primary receptor for FGF19 on fibroblasts.^[^ ^20^ ^]^ Consistent with this, our experiments confirmed that elevated FGF19 levels, as well as co‐culture conditions, upregulate FGFR4 expression on the surface of fibroblasts at the RNA and protein level (Figure S3D, Supporting Information). The differentially expressed FGF family is also validated in scRNA sequencing (Figure S3E, Supporting Information). Single‐cell transcriptomics of DEGs between MR and MS epithelial cells revealed enrichment of chemokine/MAPK signaling pathways (Table S1, Supporting Information, *p*‐value < 0.01, Figure 3D,E). GO analysis indicated similar results (Figure S3F, Supporting Information). FGF modulates chemokine signaling by upregulating CXCL12/CXCR4 axis activity, amplifying stromal‐epithelial crosstalk in MR tumors.^[^ ^21^ ^]^ Meanwhile, KEGG pathway analysis revealed that these signaling cascades (chemokine/MAPK/HIF‐1) are functionally linked to oxidative stress (ROS) responses.^[^ ^22^ ^]^ Given this association, investigating whether FGF exacerbates ProR in EC by promoting ROS production in activated fibroblasts represents a critical next step. Functional assays demonstrated that FGF19 treatment decreased glutathione (GSH) and increased malondialdehyde (MDA) and 4‐hydroxynonenal (4‐HNE) levels in fibroblast cells, confirming its ROS induction (Figure 3F). This effect was reversed by ROS inhibitors (DFO/NAC) and an FGFR4 antagonist, implicating FGF19‐driven oxidative stress as a mechanistic link to ProR. These findings collectively demonstrate that epithelial‐derived FGF19 recruits fibroblasts and induces ROS production, ultimately enhancing ProR in EC cells.

### 2.4 OxLDL‐Induced Lipid Deposition Reduces the MPA Sensitivity of EC

**Figure 4:**
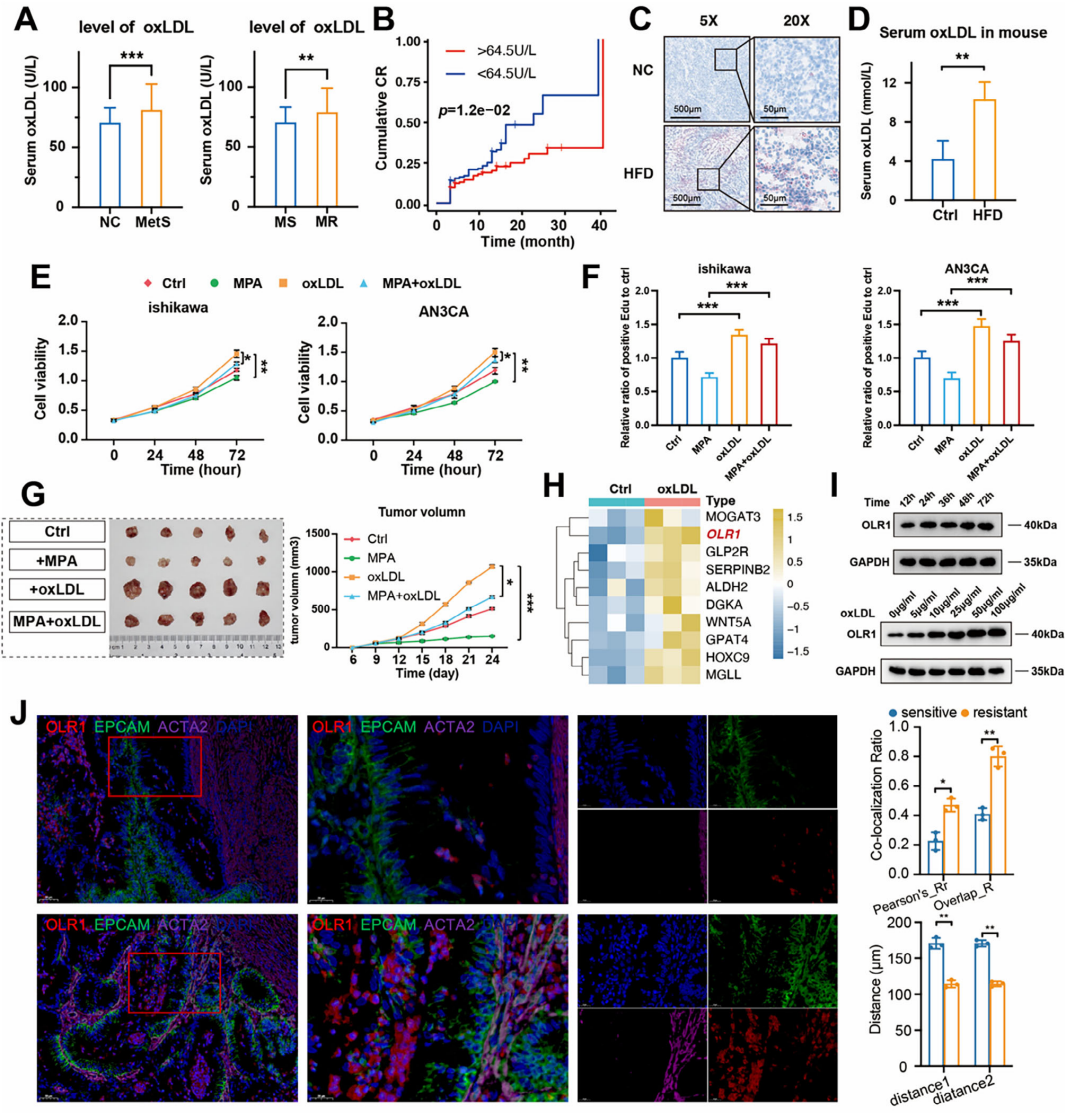
Analysis of oxLDL on EC prognosis and MPA resistance for EC. A) Level of oxLDL in 190 EC patients with/without metabolic syndrome or MPA sensitive /resistant, *p*‐values were determined using Student's t‐test. B) Cumulative CR in 190 fertility preservation EC patients with different serum oxLDL levels, adjusted by age and tumor stage. *p* values were calculated using the log‐rank test. C) Oil red staining of xenograft models in HFD or control groups (n=5 for each group). D) Serum oxLDL in different diet model groups (*n* = 5 for each group). E‐F) CCK8 and Edu assay of EC cell lines after the addition of MPA or oxLDL (50 µm). Both are treated in Ishikawa and AN3CA cells, *n* = 3 independent biological replicates. G) Representative image of tumor xenografts harvested 24 days after different treatments (*n* = 5 mice for each group). Right panel, tumor growth curves of Ishikawa xenografts after different treatments. H) Differentially expressed glycerolipid metabolism‐related genes before and after oxLDL treatment in Ishikawa cells. I) Time‐ and dose‐dependent expression of OLR1 treated with oxLDL, *n* = 3 independent biological replicates. J) Representative multiplex immunofluorescence (mIF) staining images showing the co‐localization of OLR1 and epithelial cells, and the average distance between epithelial and fibroblast cells. OLR1 (red), EPCAM (green), ACTA2 (purple), and DAPI (blue), in individual and merged channels, are shown. Spatial co‐localization was quantitatively assessed using Pearson_Rr and overlap_R. The distances from the epithelial cell to the fibroblast (distance 1) and fibroblast to the epithelial cell were calculated by center of mass. Both of the methods are from ImageJ (v1.54). All histogram and line chart data are presented as the mean ± SD. Statistical analyses were performed by Graphpad Prism 10.2.3 using the two‐tailed Student's t‐test to detect differences between the groups. Experiments were repeated at least three times. MetS, metabolic syndrome. NC, normal control. MS, MPA sensitive, MR, MPA resistant. ^*^ *p* < 0.05, ^**^ *p* < 0.01, ^***^ *p* < 0.001.

Given that fibroblast‐derived ROS promotes inflammatory cytokine secretion and protein oxidation, we investigated the role of lipid metabolism in ProR. LDL, a key mediator of lipid transport, has been implicated in tumor progression through its oxidized form (oxLDL), which enhances cancer cell proliferation and therapy resistance.^[^ ^23^ ^]^ First, oxLDL levels were compared in 190 fertility‐preserving EC patients. The patients were divided into normal or metabolic syndrome (MetS) groups and MR or MS groups, and oxLDL levels were compared between the two groups. It was confirmed that oxLDL levels were significantly increased in both MetS and MR groups (**Figure** **4**A). The complete regression (CR) rate was significantly reduced in patients with high oxLDL levels (Figure 4B). Next, to investigate the mechanism of lipid metabolic dysregulation, a mouse model of lipid metabolic disorder was established using a high‐fat diet (HFD).^[^ ^24^ ^]^ In the metabolic disorder group, significant lipid accumulation was observed (Figure 4C), and serum oxLDL levels were elevated in the HFD group (Figure 4D). Additionally, oxLDL was found to induce lipid deposition in EC cell lines (Figure S4A, Supporting Information). Previous studies have shown that lipid deposition in EC significantly reduces responsiveness to progesterone therapy.^[^ ^25^ ^]^ To validate this finding, progesterone receptor (PR) expression was analyzed in patient samples with different oxLDL levels. It was found that as both the expression level and percentage of PR positive cells increased, oxLDL levels gradually decreased (Figure S4B, Supporting Information). To further investigate the effect of oxLDL concentration on EC cell proliferation, a concentration gradient was established It was found that oxLDL concentrations above 50 µg mL^−1^ no longer exerted significant stimulatory effects on EC cell proliferation (Figure S4C–F, Supporting Information). Functionally, oxLDL attenuated the anti‐proliferative effect of MPA in EC cells (*p* < 0.01, Figure 4E,F; Figure S4G,H, Supporting Information) and accelerated tumor growth in MPA‐treated mice (*p* < 0.05, Figure 4G; Figure S4I, Supporting Information). The biological functions of oxLDL are mediated through its binding to the cell membrane receptor OLR1/LOX‐1. What's more, OLR1 is a well‐known target for oxLDL.^[^ ^26^ ^]^ Therefore, we investigated downstream OLR1 in EC. The RNA‐seq results of EC cell lines revealed that after oxLDL treatment, the expression of lipid metabolism‐related genes increased, including OLR1 (Figure 4H). Mechanistically, oxLDL upregulated its receptor OLR1 in a dose‐ and time‐dependent manner (Figure 4I; Figure S4J, Supporting Information), implicating the oxLDL‐OLR1 axis in metabolic reprogramming and ProR. To investigate whether OLR1 expression in epithelial cells is spatially proximal to fibroblasts, we performed mIF staining. As shown in Figure 4J, mIF confirmed a higher co‐localization ratio of OLR1 with epithelial cells in the MR group. Furthermore, fibroblasts and epithelial cells exhibited significantly closer spatial proximity in the MR group. Our findings position oxLDL‐OLR1 signaling as a dual mediator of metabolic rewiring and ProR in EC, with clinical evidence linking hyperlipidemia to hormonal therapy failure.

### 2.5 OLR1 Plays a Role in Reducing the Sensitivity of MPA Treatment in EC

**Figure 5:**
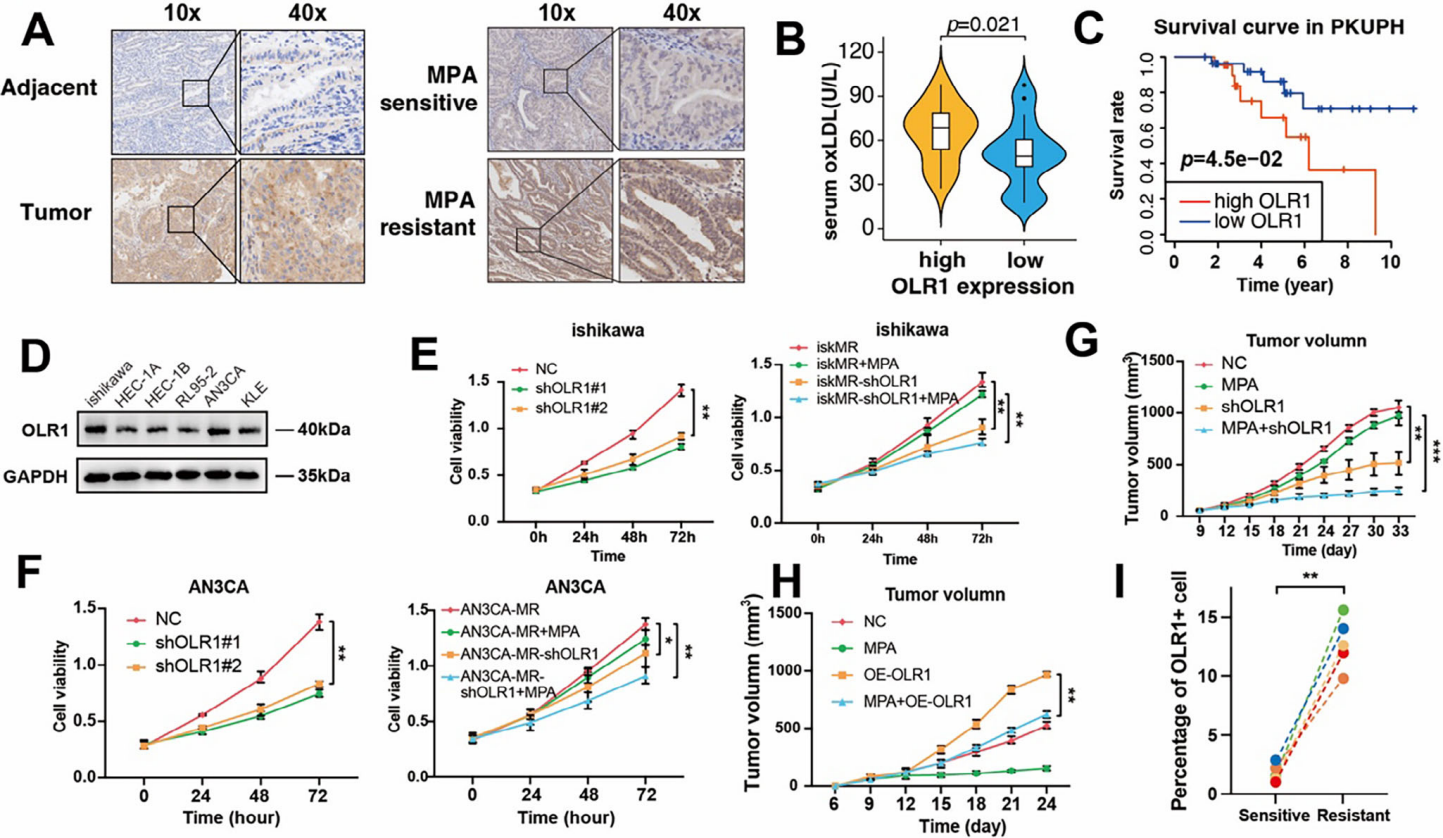
Expression and in vitro or in vivo functional validation of OLR1 in EC. A) Representative images of IHC staining of OLR1 expression in paired 10 EC tissues and their matched normal endometrium (left panel), and 10 ProS sensitive or ProR tissues. B) Comparison of the serum oxLDL level in high and low expression of OLR1 (*p* = 0.021) in 50 EC patients. C) Kaplan–Meier overall survival curves of EC patients stratified by high and low expression of OLR1 by IHC staining in PKUPH (*n* = 50), adjusted by age and tumor stage. P values were calculated using the log‐rank test. D) Protein level of OLR1 in six types of EC cell lines, *n* = 3 independent biological replicates. E, F) Cell viability assay after OLR1 deletion or over‐expression compared between the control and MPA‐resistant groups. G, H). Tumor growth curves of xenografts after transfecting with the shOLR1/OE‐OLR1 plasmid treated with or without MPA. I) Comparison of frequencies of OLR1+ epithelial cells in MPA‐sensitive (*n* = 5) and resistant (*n* = 5) tissues in scRNA‐seq. All curve chart data are collected by 3 biological replicates. ^*^ *p* < 0.05, ^**^ *p* < 0.01, ^***^ *p* < 0.001.

To elucidate the functional role of OLR1 in ProR, we systematically analyzed its clinical and molecular relevance. expression of OLR1 in 10 matched specimens revealed strong OLR1 staining in EC tissues vs adjacent non‐cancer tissues (**Figure** **5**A, left panel). Expression of OLR1 was particularly intense in MPA‐resistant cases (n = 50, Figure 5A right panel; Figure S5A, Supporting Information). A bidirectional association was observed: OLR1‐high patients displayed significantly higher serum oxLDL levels (*p* = 0.021, Figure 5B). Clinically, high OLR1 expression predicted worse overall survival in both TCGA (n = 532, *p * = 0.032, Figure S5B, Supporting Information) and PKUPH cohorts (*n* = 50, *p* = 0.045, Figure 5C). In TCGA, OLR1 expression was significantly elevated in tumor tissues compared to normal endometrium (*p *< 0.001), with further increases in lymph node metastases (*p* = 0.003) and positive peritoneal ascites (*p * = 0.007, Figure S5C, Supporting Information). Independent validation in GSE121367 and GSE17025 datasets confirmed higher OLR1 levels in progesterone‐resistant cell lines and tumor tissues (*p* < 0.05, Figure S5D, Supporting Information). Previous studies have established OLR1 involvement in tumor progression and therapy resistance across multiple cancer types, including breast and colorectal cancers.^[^ ^27^ ^]^ Mechanistically, OLR1 may attenuate drug efficacy by promoting downstream proliferation, thereby compromising tumor cell killing.^[^ ^28^ ^]^ Functional characterization across six EC cell lines identified ishikawa and AN3CA as OLR1‐high models (3.5‐ and 2.8‐fold vs RL95‐2, *p* < 0.01, Figure 5D). OLR1 knockdown in Ishikawa cells and AN3CA cells significantly impaired proliferation in both sensitive and resistant sublines (Figure 5E,F; Figure S5E, Supporting Information). Meanwhile, overexpression of OLR1 in HEC‐1A and HEC‐1B cells could significantly increase the viability of EC cells, and decreased their sensitivity to MPA (Figure S5F, Supporting Information). Xenograft models corroborated these findings, demonstrating that OLR1 depletion reduced tumor growth (*p* = 0.005) and restored progesterone sensitivity (Figure 5G,H; Figure S5G,H, Supporting Information). To investigate the role of OLR1 of epithelial cells in ProR, we re‐clustered epithelial cells from our scRNA‐seq data into six distinct subpopulations: glandular cells (FOXA2+), PGR+ cells (PGR+), luminal cells (WNT7A+), OLR1+ cells (OLR1+), oncogenic cells (LCN+), and other cells.^[^ ^16^ ^]^ Subpopulation distribution across samples revealed that OLR1+ cells were enriched in MR tumors (Figure 5I; Figure S5I, Supporting Information). CIBERSORT analysis of TCGA data confirmed elevated OLR1+ cell abundance in EC vs normal endometrium (*p* < 0.001, Figure S5J, Supporting Information), with high OLR1+ levels predicting worse overall survival (*p* < 0.001, Figure S5K, Supporting Information). Overall, these results indicate that increased expression of OLR1 in EC cells can significantly reduce sensitivity to MPA.

### 2.6 OLR1 Drives Progesterone Resistance in EC via Transcriptional Activation of FOXM1

**Figure 6:**
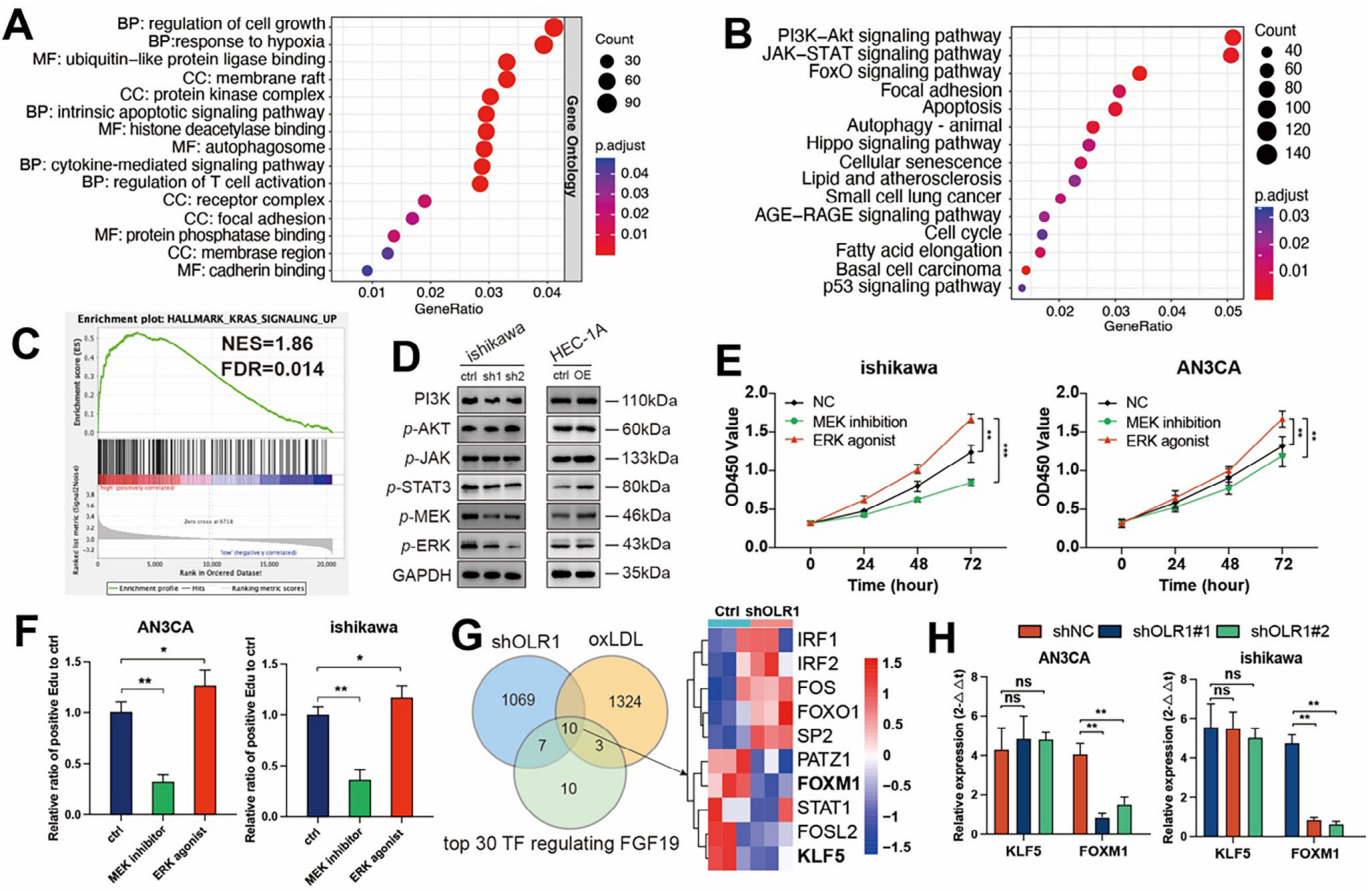
Identification and validation of the pathway and transcription factor in the downstream of OLR1. A, B) GO and KEGG analysis of DEGs between high and low expression of OLR1 with RNAseq. C) Kras signaling pathway shown by GSEA with high and low expression of OLR1 in TCGA. D) Validation of three signaling pathway proteins in shOLR1 and over‐expression of OLR1 by Western blot. E, F) Viability assay and Edu assay with MEK inhibitor or ERK agonist with Ishikawa and AN3CA, *n* = 3 independent biological replicates. G) Venn diagram and heatmap showing the common genes from three gene sets, including DEGs from shOLR1, oxLDL, and the top 30 TF from tfTarget. H) Validation of TF expression with control or shOLR1 cells at the RNA level. In all statistical plots, data are expressed as the mean ± SD Student's t‐test (Figure 6F and H) was used to determine statistical significance, performed by Graphpad Prism 10.2.3. ^*^ *p* < 0.05, ^**^ *p* < 0.01, ^***^ *p* < 0.001.

Our comprehensive investigation revealed that oxLDL and its receptor OLR1 played pivotal roles in EC progression and ProR. To investigate OLR1's downstream functions in EC, we performed transcriptome sequencing of ishikawa cells before and after OLR1 knockdown, with results shown in Figure S6A,B (Supporting Information). Functional analysis of downstream effects revealed that differentially expressed genes were primarily enriched in the regulation of cell growth, response to hypoxia, and cytokine‐mediated signaling pathway (**Figure** **6**A,B). Pathway analysis identified three key signaling pathways: KRAS signaling, JAK‐STAT signaling, and PI3K‐AKT signaling (Figure 6C; Figure S6C, Supporting Information). We validated the three significantly altered signaling pathways through Western blot. A comprehensive analysis of both the total and phosphorylated protein levels of key pathway components was conducted. This revealed that the substantial changes in the MEK‐ERK pathway activity following OLR1 manipulation were driven primarily by alterations in phosphorylation states, whereas the total protein levels remained largely unchanged (data not shown, Figure 6D), a finding further confirmed by functional experiments using ERK agonists and MEK inhibitors (Figure 6E,F; Figure S6D,E, Supporting Information). Given the susceptibility of FGF19 to upstream transcriptional regulation, we intersected three datasets (including the top 30 predicted FGF19‐associated genes from the tfTarget database) and identified 10 consensus regulators (Figure 6G). Single‐cell TF regulon analysis of OLR1+ epithelial cells highlighted KLF5 and FOXM1 among the top enriched transcription factors (Figure S6F, Supporting Information), with t‐SNE visualization showing FOXM1's distribution pattern most closely aligned with OLR1+ epithelial populations (Figure S6G, Supporting Information). It is reported that KLF5 and FOXM1 are both increased downstream of oxLDL in different diseases. Subsequent qPCR validated this observation (Figure 6H). It is reported that the stromal HAND2regulated epithelial proliferation via the ERK pathway. To validate this finding, we co‐cultured fibroblasts with EC cells before and after FGF19 knockdown. The results showed that FGF19 could significantly influence the expression of HAND2 and ERK at the mRNA level (Figure S6H, Supporting Information). These findings establish that the OLR1‐FOXM1 axis links lipid metabolism to transcriptional reprogramming, driving ProR and revealing therapeutic targets in EC.

### 2.7 FOXM1 Drives FGF19‐Mediated ProR via Transcriptional Reprogramming in EC

**Figure 7:**
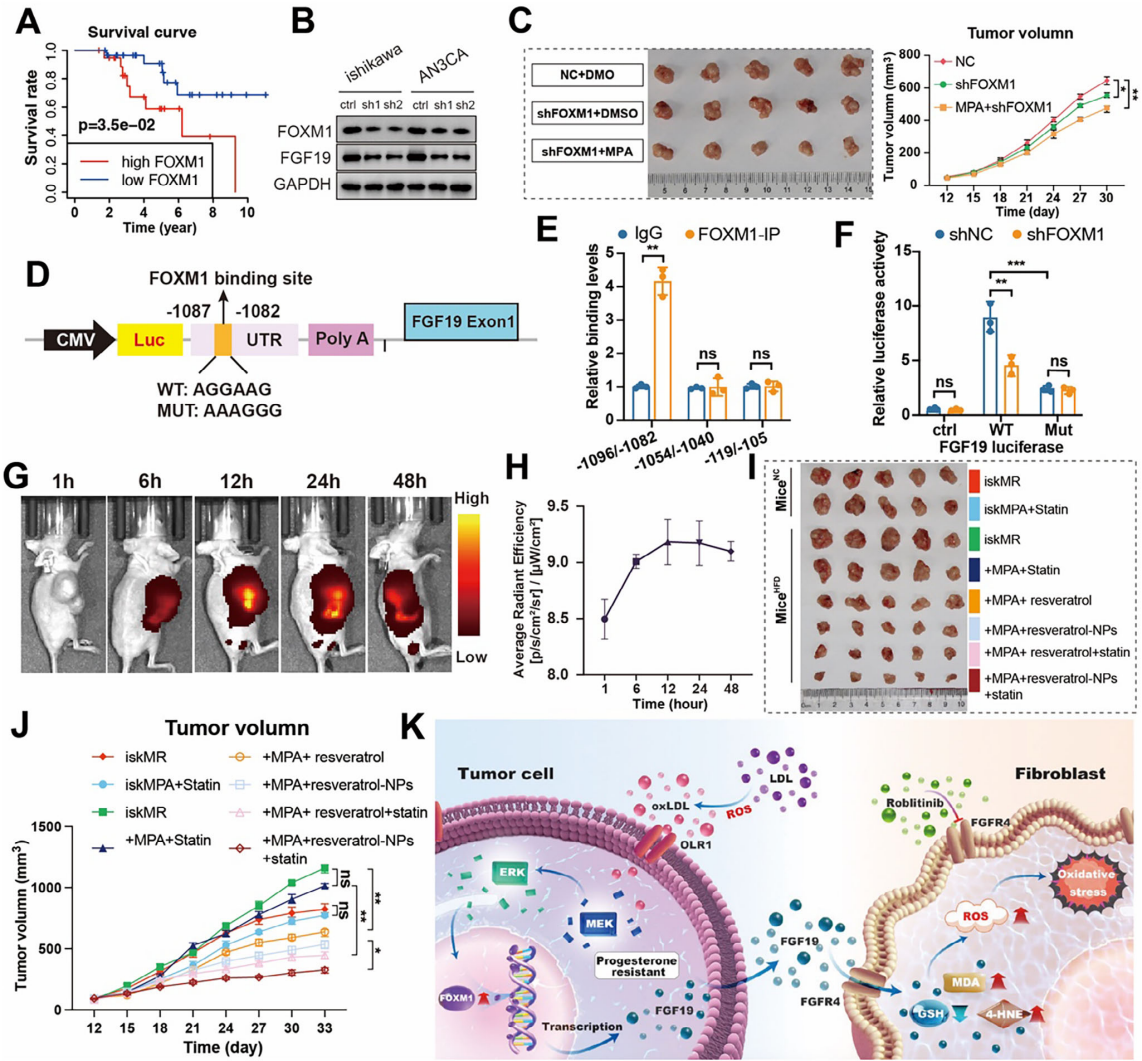
FOXM1 mediated metabolic‐transcriptional crosstalk with a positive loop between EC cancer cells and fibroblasts. A) The Kaplan–Meier overall survival curves of PKUPH patients stratified by high and low expression of FOXM1 (*n* = 49 from patients' tissues with RNAseq), statistically tested using the log‐rank test. B) Protein expression of FOXM1 and FGF19 in different expression levels of OLR1 by western blot, *n* = 3 independent biological replicates. C) Images of tumors transfected with control or shFOXM1 plasmid, and treated with MPA or DMSO. The right image shows the tumor volume of three groups of mice (*n* = 5 for each group). D) The sequences of predicted sites and the scheme of the mutations in this study the JASPAR database. E) ChIP assay showed the FOXM1 enrichment at different binding sites of the FGF19 promoter in Ishikawa cells. F) Luciferase reporter assays indicated that FOXM1 expression enhanced the FGF19 promoter‐controlled luciferase expression, which was abrogated by the site mutation in the FGF19 promoter in Ishikawa cells, *n* = 3 independent biological replicates. G) In vivo fluorescent imaging was performed for the biodistribution of RSV‐NPs@Cy7.5. RSV‐NPs were labeled with Cy7.5. H) Semiquantitative study of the fluorescence intensity of RSV‐NPs@Cy7.5 in tumor tissues at different time points. I) Images of tumor tissues isolated from mice (*n* = 5 mice for each group). J) Tumor volumes were shown. The data were shown in mean ± standard deviation (*n* = 5 for each group). One‐way ANOVA was used to analyze the differences between three or more sample groups. K) Schematic diagram illustrating the hypothetical mechanisms of CAF‐epithelial cell crosstalk in progesterone resistance of EC. Data are shown as mean ±SD. All experiments were repeated at least three times. ^*^ *p* < 0.05, ^**^ *p* < 0.01, ^***^ *p* < 0.001.

Our mechanistic dissection revealed FOXM1 as the critical transcriptional effector downstream of OLR1, orchestrating FGF19‐driven ProR. Clinically, elevated FOXM1 expression correlated with worse overall survival in both TCGA and PKUPH cohorts (*p* <0.05) (**Figure** **7**A; Figure S7A, Supporting Information), while correlative analysis demonstrated that FOXM1 is positively associated with FGF19 (R = 0.34, *p* <0.001) (Figure S7B, Supporting Information). FOXM1 expression demonstrated high predictive accuracy for judging EC (AUC = 0.98, Figure S7C, Supporting Information). Genetic validation showed that OLR1 knockdown concurrently reduced FOXM1 and FGF19 levels (Figure 7B), positioning FOXM1 as a mediator of OLR1's effects. Functional role of FOXM1 aligns with its established oncogenic functions in breast and lung cancer, where it promotes therapy resistance through ubiquitination of TOP2A to induce senescence and modulates PD‐L1 expression and cell proliferation.^[^ ^29^, ^30^ ^]^ In EC models, FOXM1 depletion suppressed proliferation and restored progesterone sensitivity in vivo (Figure 7C; Figure S7D, Supporting Information) and in vitro (Figure S7E–G, Supporting Information). To establish direct transcriptional regulation, we engineered FGF19 promoter mutants (WT vs MUT at −1087/−1082 bp, Figure 7D). ChIP analysis also confirmed FOXM1 binding to the FGF promoter(Figure 7E), confirming FOXM1 as a bona fide FGF19 transcription factor. Luciferase assays demonstrated that FOXM1's transactivation of FGF19 was abolished in the mutant construct (*p* = 0.007 vs WT, Figure 7F). Resveratrol (RSV), which exerts antioxidant effects in tumors, is gaining increasing attention for its therapeutic and tumor‐suppressive properties in cancer.^[^ ^31^ ^]^ However, its potential to enhance progesterone sensitivity in endometrial cancer has not yet been investigated. To improve its targeting to tumor tissues, RSV was encapsulated with nanoparticles (RSV‐NPs). Next, we constructed a subcutaneous HFD EC tumor‐bearing mice model (miceEC+/HFD+) to evaluate RSV‐NPs combined with HFD in vivo. First, to observe the accumulation of RSV‐NPs, RSV‐NPs were labeled with Cy7.5 and injected intravenously. The in vivo imaging showed that the fluorescence signal gradually increased at the tumor site over time and reached its maximum at 12 h (Figure 7G,H). A schematic diagram of this study is shown in Figure S7H (Supporting Information). Therapeutic validation in HFD‐fed mice showed that lipid metabolic reprogramming exacerbated tumor growth and ProR, which was reversed by statin/RSV‐NP combination therapy (Figure 7I,J; Figure S7I, Supporting Information). A schematic illustration of this study is shown in Figure 7K. These results reveal that lipid metabolic reprogramming promotes ProR through the OLR1‐FOXM1‐FGF19 axis, while nanoparticle‐encapsulated resveratrol restores hormonal sensitivity and suppresses tumor growth in vivo. Our findings identify novel therapeutic targets and support metabolic interventions to overcome treatment resistance.

## 3 Discussion

In this study, we investigated the differences in the tumor microenvironment between progesterone‐sensitive and progesterone‐resistant groups using scRNA data. Unsupervised clustering identified 19 distinct cell subpopulations, which were annotated based on canonical markers. Compared to the sensitive group, resistant lesions displayed a significantly higher proportion of fibroblasts and epithelial cells. We propose that dysregulated lipid metabolism in epithelial cells leads to increased secretion of FGF19, which in turn exacerbates ROS in fibroblasts. Heightened ROS in fibroblasts promotes the conversion of LDL into oxLDL within the tumor microenvironment. OxLDL then binds to its receptor OLR1 on epithelial cells, further amplifying lipid metabolic disturbances and reinforcing FGF19 production. Together, these processes form a positive feedback loop between epithelial cells and fibroblasts, driving progesterone resistance in EC. In addition, other metabolic pathways, such as steroid hormone biosynthesis, were also significantly enriched. Although not mechanistically explored here, its upregulation may reflect adaptive hormonal reprogramming contributing to therapy resistance, representing a compelling avenue for future investigation into EC progression.

Our findings reveal the OLR1/FOXM1/FGF19 axis as a central metabolic‐transcriptional hub linking lipid uptake to progestin resistance. In oxLDL‐rich microenvironments, OLR1 activates MAPK signaling and upregulates FOXM1, which directly binds and transactivates the FGF19 promoter.^[^ ^32^ ^]^ This establishes a feed‐forward loop that sustains proliferation and dampens the MPA response. Beyond biomass supply, lipid metabolic rewiring epigenetically reinforces this program: oxLDL‐derived acetyl‐CoA promotes histone acetylation, enhancing chromatin accessibility at the FOXM1 and FGF19 loci and locking cells into a therapy‐resistant transcriptional state.^[^ ^33^ ^]^

Given the global obesity pandemic, the metabolic underpinnings of EC progression have garnered increasing attention. Among these, the OLR1/FOXM1/FGF19 signaling axis has emerged as a potential driver of tumor‐stroma interactions in metabolically dysregulated environments. Previous studies have identified OLR1 as a key sensor of oxLDL, linking lipid metabolic stress to pro‐tumorigenic signaling cascades, particularly in obesity‐associated cancers.^[^ ^34^ ^]^ In breast and pancreatic cancers, OLR1 activation has been shown to induce FOXM1‐mediated transcriptional programs that promote cell proliferation and resistance to therapy,^[^ ^9^, ^35^ ^]^ suggesting that OLR1 may function as a broader metabolic‐stress integrator across malignancies. Meanwhile, FGF19‐traditionally implicated in bile acid and glucose homeostasis been increasingly recognized for its paracrine role in the tumor microenvironment.^[^ ^36^ ^]^ It can activate stromal fibroblasts via FGFR1 signaling, contributing to ECM remodeling and pro‐invasive phenotypes.^[^ ^37^ ^]^ Several reports have demonstrated that FOXM1 directly regulates FGF19 transcription in hepatocellular and colorectal cancers, forming a positive feedback loop that reinforces metabolic reprogramming and stromal activation.^[^ ^38^ ^]^ These findings collectively suggest that the OLR1/FOXM1/FGF19 axis may represent a critical regulatory circuit linking lipid‐driven epigenetic reprogramming withfibroblast activation and stromal remodeling. Targeting this axis could offer novel therapeutic avenues, particularly in obese EC patients where metabolic cues strongly influence disease trajectory.

FOXM1 functions as a master transcriptional regulator that integrates metabolic and developmental signals to modulate tumor progression.^[^ ^39^ ^]^ Importantly, FOXM1 preferentially binds the FGF19 promoter, driving its expression and linking transcriptional control to intercellular signaling.^[^ ^40^, ^41^ ^]^ Members of the FGF family are known to promote tumor proliferation and endocrine resistance; our findings reinforce this by showing that FGF19 not only supports epithelial cell survival but also initiates fibroblast activation via paracrine signaling. Notably, this activation is temporally phased‐initially driven by FGF19 and sustained by ROS production‐creating a self‐reinforcing tumor‐promoting microenvironment. Furthermore, FOXM1 is well‐established as a key regulator of tumor metabolic reprogramming, iron metabolism, and metastasis across multiple cancer types,^[^ ^42^ ^]^ reinforcing its central role in endometrial carcinogenesis. Collectively, our study positions FGF19 as a critical bridge between transcriptional reprogramming and microenvironmental remodeling, and highlights FOXM1 as a potential upstream vulnerability with broad implications for endocrine resistance.

FGF family plays multifaceted roles in shaping the gynecologic tumor microenvironment, not only by enhancing tumor cell proliferation, but also by reprogramming stromal components.^[^ ^43^ ^]^ Our data demonstrate that tumor‐derived FGF19 serves as a key initiator of fibroblast activation, inducing α‐SMA expression and ECM remodeling within 48 h. This early activation is followed by a maintenance phase characterized by elevated ROS levels, which are sustained through metabolic stress and further fuel fibroblast reactivity.^[^ ^44^ ^]^ Importantly, ROS not only perpetuates fibroblast activation, but also convert LDL into oxLDL.^[^ ^45^ ^]^ This creates a vicious cycle wherein FGF19‐induced fibroblast activation elevates ROS, generating oxLDL that further stimulates OLR1‐FOXM1 signaling in epithelial cells, promoting progesterone resistance and proliferation. This ROS‐lipid‐transcriptional loop highlights a previously underappreciated metabolic‐stromal axis in EC. The coupling of oxidative stress and lipid signaling offers a mechanistic explanation for the sustained tumor‐supportive state of fibroblasts and suggests that antioxidant or lipid‐targeting therapies may synergize with endocrine treatments to disrupt this pathogenic feedback. Furthermore, FGF19 mediates critical epithelial‐stromal crosstalk that fosters a treatment‐refractory niche. Epithelial‐derived FGF19 activates fibroblasts—potentially inducing CAF transformation—which in turn promotes ECM remodeling, LDL oxidation, and feedback via FGFR‐SRC/MAPK signaling to cancer cells.^[^ ^46^ ^]^ This aligns with established stroma‐mediated resistance mechanisms, such as HGF/MET‐driven tolerance in melanoma or IL‐6/STAT3‐induced chemo‐resistance in colorectal cancer.^[^ ^47^ ^]^ Targeting this axis may require combinatorial strategies co‐inhibiting FGFR and downstream effectors, though compensatory signaling via alternative receptors or cytokines remains a potential challenge.

Despite the strengths of our integrative approach, several limitations should be acknowledged. First, while our scRNA and experimental data support the OLR1–FOXM1–FGF19 axis in fibroblast–epithelial crosstalk, in vivo validation through lineage tracing or conditional knockouts remains necessary. Second, our findings are largely based on resistant EC models and may not fully capture dynamics in hormone‐sensitive disease. Third, although ROS was implicated as a driver of oxLDL formation, the upstream regulation and the cellular source of oxidative stress in fibroblasts and the microenvironment require further clarification. Future studies should explore metabolic flux analysis and in vivo perturbation models to dissect these pathways more precisely.

In conclusion, we have identify the OLR1/FOXM1/FGF19 axis as a key driver of progesterone resistance in EC. Lipid metabolic dysregulation in epithelial cells and oxidative stress in fibroblasts form a positive feedback loop via oxLDL‐OLR1 signaling, suggesting that targeting ROS or lipid pathways may improve the sensitivity to progesterone therapy for EC patients.

## 4 Experimental Section

### Cell Lines and Reagents

The EC cell lines, including Ishikawa (RRID: CVCL_2529), AN3CA (RRID: CVCL_5521), and Ishikawawere obtained from laboratory stocks in Peking University People's Hospital (PKUPH) and cultured at 5% CO_2_ and 37 °C in DMEM/F12 medium (Gibco, Invitrogen, Carlsbad, CA, USA) supplied with 10% fetal bovine serum (Gibco, Invitrogen). They were also tested negative for mycoplasma contamination and authenticated by STR profiling. AN3CA cells were routinely grown in McCoy's 5A media at 37 °C in a 5% CO_2_ humidified atmosphere. The detailed methods of culturing cells have been well described in the previous study. MPA was used to establish an MPA‐resistant (MR) ishikawa cell line, which ishikawaMR (or ishMR) was referred to as; this cell line was created via the increasing concentration gradient method. 10 µm MPA was also added to the medium containing the ishMR cells to maintain resistance. For this study, the following antibodies were purchased against to OLR1 (Proteintech, 11837‐1‐AP), PI3K (CST, #4249), p‐AKT (CST, # 4060), p‐JAK (Proteintech, 29101‐1‐AP), p‐STAT3 (CST, #9145), p‐MEK (CST, #9154), p‐ERK (CST, #3179), FOXM1 (Proteintech, 13147‐1‐AP), FGF19 (abcam, ab320829), PGR (CST, #8757), FGF1 (Proteintech, 17400‐1‐AP), FGF9 (Proteintech, 26554‐1‐AP), FGF13 (abcam, ab186300), and GAPDH (CST, #5174). Medroxyprogesterone acetate (ab142633) and oxLDL (IO1300) were obtained from Abcam and Solarbio, respectively, and MPA was diluted in DMSO. ERK agonist (Ro 67‐7476), Roblitinib (FGF401), and MEK inhibitor (AZD6244) were purchased from MedChemExpress and Selleck, respectively.

### Patients and Clinical Specimens

All the selected patients were consecutively enrolled according to the following criteria: 1) Clinical stage I, Grade 1, endometrioid endometrial cancer (EEC); 2) Conform to fertility preservation; 3) Comprehensive clinicopathological and follow‐up information available. The patients were excluded if they had other malignant tumors before, or histories of adjuvant or neo‐adjuvant therapies, including targeted therapies. Informed consent on the use of clinical specimens was obtained from all patients. Finally, 190 patients were enrolled. The study was authorized by the Ethics Committee of Peking University People's Hospital (2022PHB397‐001). After hysteroscopy, resected tumor samples were obtained and snap‐frozen in liquid nitrogen in less than 30 min after resection in PKUPH between January 2022 and December 2024, and 49 samples were sent to RNA sequencing. The detailed clinical characteristics of the included patients are provided in Table S2 (Supporting Information). Progesterone‐resistant patients are defined as meeting any of the following criteria: 1) progressive disease at any time during conservative treatment; 2) stable disease persisting after 7 months of first‐line therapy; 3) Failure to achieve complete regression (CR) after 10 months of first‐line therapy.

### Cell Viability Assay

Cell viability was assayed by a Cell Counting Kit‐8 (CCK8) kit (Dojindo Laboratories, CK04). According to the protocol of the CCK‐8 assay, tumor cells were plated in a 96‐well plate at 3 × 10^4^ cells per well. Six parallel wells were used for each independent group. Subsequently, 100 µL of fresh medium was added to cells containing 10 µL of CCK‐8 solution and incubated for 2 h (37 °C, 5% CO_2_). Absorbance at 450 nm was measured using a microplate reader.

### EdU Incorporation Assays

A 5‐ethynyl‐20‐deoxyuridine (EdU) incorporation assay kit (Beyotime, Shanghai, China) was used to test the proliferative ability of cells. This kit was used in accordance with the manufacturer's instructions. Cells were plated in six‐well plates at a density of 2×10^5^ cells each well. Then, fresh medium was added containing specific concentrations of different regents (including MPA, oxLDL, ERK agonist, and MEK inhibitor). After 48 h, cells incubated with 10 µm EdU working solution at 37 °C for 2 h were fixed with 4% PFA for 15 min according to the manufacturer's instructions. The EdU solution was added to culture, followed by the staining of nuclei with 4′,6‐ diamidino‐2‐phenylindole (DAPI; Beyotime, Shanghai). Images were collected under a fluorescence microscope.

### Transwell Migration Assay

HESC cells were seeded on a transwell chamber with 8.0 µm pores at 1 × 105 cells per well in serum‐free medium. Then, 500 µL of cancer cell‐CM was added to the lower chamber. Recombinant FGF19 (R&D Systems) was diluted in serum‐free medium to a final concentration of 50 ng mL^−1^. After incubation for 12 h, cells in the transwell chamber were fixed with methanol and stained with H&E. The non‐migrated cells were removed with a cotton swab, and the migrated cells were visualized under a phase‐contrast microscope and manually counted in three randomly selected fields (magnification, ×100).

### Oil Red O Staining

Paraffin‐embedded tissue sections were dewaxed, rehydrated, and rinsed with distilled water in human tissue samples and mouse tumor samples. After immersion in 60% isopropanol for 5 min, sections were stained with Oil red O solution (G1260, Solarbio Life Sciences, Beijing, China) for 15 min according to the manufacturer's instructions, followed by differentiation in 60% isopropanol. Nuclei were counterstained with hematoxylin. Slides were mounted with glycerin jelly and imaged under a light microscope to evaluate lipid deposition.

### ELISA Assays

Glutathione (GSH, Beyotime, S0053), Malondialdehyde (MDA, Beyotime, S0131S), and 4‐Hydroxynonenal (4‐HNE, Salmart, SE‐KTE4051) levels in cell culture supernatants were quantified using a commercial ELISA kit following the manufacturer's protocol. Briefly, supernatants were centrifuged (12 000 rpm, 10 min, 4 °C) to remove debris, and absorbance was measured at 450 nm. For oxLDL analysis in serum samples, a Share‐Bio ELISA kit (SB‐FY7076) was employed. Serum was diluted as specified, and optical density (OD) was determined at 450 nm using a microplate reader. Standard curves were generated for each assay to calculate concentrations. All samples were run in duplicate, and data were normalized to total protein or volume as appropriate.

### Preparation and Characterization of Nanoparticles (resveratrol‐NPs)

The as‐synthesized polymer P1 (10 mg) and resveratrol (1 mg) were initially dissolved in 1 mL of DMSO, and then the solution was dispersed in 10 mL of de‐ionized water (43 °C) with continuous stirring. After vigorous stirring for 15 min, the mixture was collected and dialyzed using a dialysis bag (MWCO: 3500 Da) overnight. The nanoparticles (termed resveratrol‐NPs hereafter) were then separated by centrifugation and washed twice with deionized water. The morphology of resveratrol‐NPs was characterized by TEM (HT‐7700, Hitachi, Japan). The size of resveratrol‐NPs was measured by a Malvern Zetasizer Nano ZS90 laser particle size analyzer (Nano ZS, UK).

### In Vivo Imaging and Biodistribution Analysis

Ishikawa cells (3 × 10^6^) were subcutaneously injected into the hip of female BALB/c mice. When the tumor volumes reached ≈1000 mm^3^, mice were i.v. injected with RSV‐NPs@Cy7.5. After injection, the fluorescence signals were recorded on the IVIS spectrum imaging system (Spectrum CT, PerkinElmer) at 1, 6, 12, 24, 48 h.

### Single‐Cell Sequencing

Tissues were collected and dissociated into single‐cell suspensions by enzymatic digestion with collagenase I/IV and DNase I, the first time they were diagnosed, followed by filtration through 40–70 µm strainers and red blood cell lysis. In the present cohort, the majority of patients presented with abnormal uterine bleeding and underwent diagnostic curettage outside of a regular menstrual cycle. Moreover, subsequent high‐dose progestin therapy effectively suppressed physiological endometrial cycling, thereby minimizing confounding effects from natural hormonal fluctuations. Cell viability was assessed using acridine orange/propidium iodide staining, and dead cells were removed using MACS kits. Single‐cell libraries were prepared using the 10× Genomics Chromium Controller (Single Cell 3′ v3 Reagent Kits), targeting 3000–8000 cells per sample. Gel bead‐in‐emulsions (GEMs) were generated, followed by cDNA synthesis, amplification, and library construction. Libraries were quantified (Qubit HS DNA assay) and sized (Bioanalyzer 2200) before paired‐end sequencing (150 bp) on Illumina platforms (NovaSeq 6000). Raw sequencing data were processed using CellRanger (GRCh38 reference genome). Quality control in Seurat (v4.3.0) excluded cells with <2000 or >30000 UMIs, >6000 genes, or >20% mitochondrial reads. Doublets (7.6%) were removed via DoubletFinder. Datasets were integrated using “FindIntegrationAnchors” (3000 features, 20 dimensions) and Harmony. Clustering and marker identification (“FindAllMarkers”) enabled cell‐type annotation. Pathway analysis was performed using GSEA.

### Comprehensive scRNA‐Seq Data Preprocessing

DoubletFinder identified and removed 7.6% doublets, resulting in a final dataset of 108557 cells and 32514 genes for downstream analysis. To ensure data consistency across samples, integration was performed using Seurat's IntegrateData function followed by Harmony‐based batch correction. Dimensionality reduction was achieved through PCA (utilizing the top 2000 highly variable genes) and visualized using UMAP. Unsupervised clustering (FindNeighbors/FindClusters, resolution = 1.0) revealed distinct cell populations that were annotated based on established marker genes: epithelial cells (EPCAM, CDH1, KRT8), fibroblasts (COL1A1, COL1A2, LUM), proliferative cells (MKI67, TOP2A, CENPF), macrophages (LYZ, CD68, CD163), endothelial cells (PECAM1, CDH5, VWF), smooth muscle cells (TAGLN, MCAM, BGN), T cells (CD3D, CD3E, CD3G), and NK cells (NKG7, KLRD1, PTPRC). Differential gene expression analysis was performed using the Wilcoxon rank‐sum test with stringent thresholds (|log2FC| > 0.25, adjusted *p*‐value < 0.05). Functional enrichment analysis of differentially expressed genes was conducted using Metascape, revealing cell‐type‐specific biological pathways. This comprehensive analytical approach provided a detailed characterization of cellular heterogeneity and transcriptional profiles within the tissue microenvironment.

### Construction of Cell–Cell Communication Networks

In order to investigate the intricate communication network among various cell types, a comprehensive cell–cell communication analysis was performed using the “CellChat” R package. The estimation of communication probabilities was achieved by integrating the gene expression matrix with existing knowledge regarding interactions among signaling ligands, receptors, and their cofactors. The interactions of ligand‐receptor pairs with a *p*‐value less than 0.05 were retained. This integrated approach provided a holistic understanding of the intricate communication landscape between different cell types within the biological system.

### Immune Cell Infiltration Analysis using CIBERSORT

To investigate the immune cell composition within EC tissues, the CIBERSORT algorithm was utilized to estimate the relative proportions of tumor‐infiltrating immune cells based on bulk RNA sequencing data. Hallmark gene sets were downloaded from The Molecular Signatures Database (MSigDB, http://software.broadin‐stitute.org/gsea/msigdb/). Gene expression profiles from 49 EC samples were analyzed using the LM22 signature matrix, which enables the deconvolution of 22 distinct immune cell subsets, including various populations of T cells, B cells, natural killer (NK) cells, macrophages, dendritic cells, and others. The analysis was conducted using the R implementation of CIBERSORT. For RNA‐seq data, quantile normalization was disabled as recommended. The number of permutations for significance analysis was set to 500 to ensure robust estimation of p‐values for each sample. Only samples with CIBERSORT output *p*‐values < 0.05 were retained for downstream analysis to ensure high‐confidence immune cell composition estimates. Based on the inferred immune cell profiles, patients were stratified into two groups according to the relative abundance of epithelial cells and fibroblast‐identified via prior annotation or supplementary computational estimates‐and performed survival analysis to explore their association with clinical outcomes. To evaluate the abundance of OLR1⁺ epithelial cells in bulk RNA‐seq samples from TCGA‐UCEC, CIBERSORTx was applied with a custom epithelial reference derived from the single‐cell data. Patients were stratified into OLR1‐high and OLR1‐low groups according to the median estimated fraction of OLR1⁺ epithelial cells, and associations with survival were analyzed using Kaplan–Meier curves and multivariate Cox regression adjusted for FIGO stage and tumor grade.

### Identification of OLR1⁺ Epithelial Cells and Deconvolution of Bulk RNA‐Seq Data

Single‐cell RNA‐seq data were processed using Seurat v4.3. Cells with >200 detected genes and <10% mitochondrial reads were retained. Gene expression was log‐normalized (NormalizeData, scale factor = 10 000) and scaled with ScaleData. Highly variable genes were identified using FindVariableFeatures (*n* = 2000). Principal component analysis was performed, and cell clustering was conducted with FindNeighbors and FindClusters (resolution = 0.6). UMAP was used for dimensionality reduction. Epithelial cells were annotated according to canonical markers (EPCAM, KRT8/18) based on CellMarker and published epithelial signatures.

### Bioinformatics Analysis in The Cancer Genome Atlas and Gene Expression Omnibus Datasets

Transcriptome expression profiles and clinical data of 532 EC patients were downloaded from the TCGA database by XENA (https://xenabrowser.net/datapages/). OLR1 expression was analyzed across different clinicopathological features and stratified by optimal cutoff using the “maxstat” R package (https://cran.r‐project.org/web/packages/maxstat/). Survival analysis was performed using the “survival” and “survminer” R packages. For external validation, OLR1 expression was compared between progesterone‐resistant and ‐sensitive EC cell lines (*n* = 3 per group) in GSE121367, and between normal endometrium (*n* = 12) and EC tissues (*n* = 91) in GSE17025, using GEOquery (https://bioconductor.org/packages/release/bioc/html/GEOquery.html) for data acquisition. GSEA, using the JAVA program (http://software.broadinstitute.org/gsea/index.jsp), was employed for assessing the possible mechanisms between high‐ and low‐risk groups based on the KEGG (v7.1, https://www.gsea‐msigdb.org/gsea/downloads.jsp) gene set collections. The number of random sample permutations was set at 1000, and | NES | > 1, NOM *p*‐value < 0.05, and FDR *q*‐value < 0.25 were set as the significance threshold. Gene Ontology (GO) analysis that includes molecular function (MF), cell component (CC), and biological process (BP) analyses was implemented to explore the possible molecular mechanisms behind ROS‐related DEGs via the clusterProfiler package of R, utilizing the same approach for KEGG analysis and considering *p* < 0.05 as significant enrichment.

### Analysis of TFs Activity and Expression

PySCENIC was used to perform the single‐cell regulatory network inference and clustering (SCENIC), which identified the specific assessment of TFs activity by the co‐expression patterns of genes. Essentially, within pySCENIC, TFs activity was evaluated by analyzing gene co‐expression patterns within individual cells, particularly focusing on genes associated with TFs. This process identified genes co‐expressed with specific TFs within individual cells, allowing inference on the activity status of these TFs. This analytical approach utilized correlations and expression patterns of genes to infer potential activity and regulatory networks of TFs within individual cells. PySCENIC used the count matrix of expression levels as input to calculate the co‐expression modules and evaluated the weight between TFs and their target genes, in which the GRNBoost algorithm was chosen. Then, TFs with direct targets were identified using the RcisTarget, and the activity of each regulon in each cell was evaluated using AUCell. Outputs of pySCENIC were input to calculate the average regulon activity (AUC) scores using “AUCell” and “SCENIC” R packages.

### Immunohistochemistry Staining and Evaluation

Immunohistochemistry (IHC) staining was performed on formalin‐fixed, paraffin‐embedded (FFPE) EC tissue sections representing different clinicopathological subtypes. Sections were cut at 5 µm thickness and processed using standard deparaffinization, rehydration, and antigen retrieval procedures. The primary antibody against OLR1 was used at a dilution of 1:200 (Abcam, ab60178), and staining was carried out following the UltraVision Quanto Detection System protocol (Thermo Fisher Scientific) with 50 clinical samples. Hematoxylin was used for nuclear counterstaining. Images were acquired using a Nikon Eclipse Ti‐s microscope (Nikon, Tokyo, Japan). For semiquantitative analysis, staining intensity (scored 0–3) and frequency of positive cells (scored 0–100%) were evaluated independently by two pathologists. A composite expression score (CES) was calculated by multiplying the intensity and frequency scores, resulting in a total range from 0 to 15. Based on prior validation, CES <7 was defined as low OLR1 expression, and CES ≥7 as high expression. CES values were compared across different histological grades and molecular subtypes of EC to assess expression patterns of OLR1.

### RNA Extraction, Reverse Transcription, and Quantitative PCR (RT‐qPCR)

Briefly, the total RNA of tissue or cancer cells was isolated using the TRIzol Reagent (Invitrogen) according to the manufacturer's protocol. One microgram of total RNA was reverse‐transcribed using the PrimeScriptTM RT reagent Kit with gDNA Eraser. The DyNAmo Color Flash SYBR Green qPCR kit (Thermo Scientific) was used to perform quantitative real‐time PCR using the Bio‐Rad machine, using the FOXM1 primers (forward, 5′‐ATACGTGGATTGAGGACCACT‐3′; reverse, 5′‐TCCAATGTCAAGTAGCGGTTG‐3′), KLF5 primers (forward, 5′‐ATACGTGGATTGAGGACCACT‐3′; reverse, 5′‐TCCAATGTCAAGTAGCGGTTG‐3), and the experimental results were analyzed.

### Western Blot Analysis

The expression of proteins was detected by Western blot. Cell lysate (RIPA: protease inhibitor: phosphorylated protease inhibitor = 100:2:1) was added to the cell dish to extract all proteins in the cell. The specific steps are as described in the previous research. The summary is as follows: protein was quantified by the Coomassie brilliant blue protein quantitation method. An appropriate separation gel was selected according to the molecular weight. 40 µg of protein sample was added per lane, and the samples were separated by SDS‐PAGE gel electrophoresis. Then the protein was transferred to the NC membrane. 5% milk or 5% BSA was used for blocking for 1 h. Antibodies were diluted with diluents at 1000:1 and incubated at 4 °C overnight. The next day, after washing the NC membrane, the secondary antibody was incubated, and the scanning quantitative analysis was carried out on an Odyssey infrared fluorescence scanning imaging system.

### Luciferase Reporter Assay

To validate the transcriptional regulation of FGF19 by FOXM1, the wild‐type (*FGF19‐WT*) and mutant (*FGF19‐MUT*) promoter regions containing the predicted FOXM1 binding sites were synthesized and cloned into the pGL3‐Basic luciferase reporter vector (Promega, Madison, WI, USA). Ishikawa cells were seeded in 24‐well plates (1.5 × 10⁵ cells per well) and co‐transfected with *FGF19‐WT* or *FGF19‐MUT* reporter plasmids, 0.08 µg of pRL‐TK renilla luciferase plasmid, and either FOXM1‐targeting shRNA plasmid (shFOXM1) or control shRNA using Lipofectamine 2000 (Invitrogen, USA). After 48 h, luciferase activity was measured using the Dual‐Luciferase Reporter Assay System (Promega, USA). Firefly luciferase activity was normalized to renilla luciferase activity.

### Chromatin Immunoprecipitation (ChIP) Assay

Cells were exposed to a 1% formaldehyde solution at room temperature for 10 min to facilitate the capture of protein‐DNA crosslinks. After washing and lysing the cells, chromatin was fragmented through sonication to achieve smaller DNA pieces. The chromatin was then incubated with anti‐FOXM1 antibody or control IgG overnight at 4 °C. The antibody‐protein‐DNA complexes were pulled down using ChIPGrade Protein G Agarose Beads (Beyotime, P2080S) and washed several times with high‐salt buffer to remove non‐specific bindings. To analyze FOXM1 binding to the FGF19 promoter, the enriched DNA was purified and subjected to quantitative PCR (qPCR) to quantify the amplification of FGF19 promoter regions. The primers for qPCR were detailed in Table S3 (Supporting Information). The relative binding of FOXM1 to the FGF19 promoter was normalized to the input and IgG control samples.

### Animals Study

All animal experiments were performed in accordance with the Guide for the Care and Use of Laboratory Animals and approved by the Animal Care and Use Committees at the Model Animal Research Center at Peking University People's Hospital (Beijing, P. R. China). Female Balb/C nude mice (4–6 weeks, 18–20 g) were purchased from Charles River (Beijing, China) and were maintained in specific pathogen‐free (SPF) facilities. A total of 1 × 10^7^ living cells with a volume of 200 µL were subcutaneously injected into the right flank of the mice. When the tumor diameter reached 5 mm, the mice were then randomly divided into different groups (*n* = 5 mice group) to receive an intraperitoneal injection of either vehicle (control), MPA (100 mg kg^−1^ bodyweight^−1^), oxLDL (5 mg kg^−1^ bodyweight^−1^, with oxLDL concentration of 5 mg mL^−1^) or MPA plus oxLDL were given every 2 days. Tumor diameters were measured three times a week using a vernier caliper, and the volume was calculated by 0.5^*^length^*^width^2^. After treatment, the mice were sacrificed, and the tumor weight was measured. After that, the tumors were sliced and stained with corresponding antibodies for immunohistochemistry. To induce metabolic dysfunction, a cohort of female BALB/c nude mice (5–6 weeks old, *n* = 5 per group, weighing 20–25 g) was fed a high‐fat diet (HFD; containing 60%kcal from fat) for a duration of 5–6 weeks. Age‐matched control mice were maintained on a standard normal chow diet (NCD; 10%kcal from fat) for the same period. Body weight and tumor volume were monitored every 3 days. The animal experiment was approved by the ethics committee of Peking University People's Hospital (2020PHE093), and the ethical requirements of experimental animals and the animal welfare law were strictly adhered to during the experimental operations.

### Statistical Analysis

Statistical analyses were conducted using GraphPad Prism version 10.2.3 and R software (version 4.4.2). The assays were repeated at least three times, and data are presented as mean ± standard deviation (SD). Student's t‐test, Wilcoxon rank‐sum test, and log‐rank test were employed for statistical analysis. One‐way ANOVA was used to analyze the differences between three or more sample groups. Detailed data on the results of all analyses are available in the Figure legends. *P*‐values < 0.05 were considered statistically significant (ns, *P* ≥ 0.05; ^*^, *P* < 0.05; ^**^, *P* < 0.01; ^***^, *P* < 0.001).

### Ethics Approval and Consent to Participate

All methods in this study were carried out in accordance with the Declaration of Helsinki, and informed consent was obtained from all patients. The authors are accountable for all aspects of the work in ensuring that questions related to the accuracy or integrity of any part of the work are appropriately investigated and resolved. The study was approved by our Institutional Review Board (Approval number: 2022PHB379‐001).

## Conflict of Interest

The authors declare no conflict of interest.

## Author Contributions

X.L. and Y.Q. contributed equally to this work. X.L., Y.Q., and Y.W. conceived and supervised the project., X.L. and J.W. designed and performed the research. Y.Q., Y.W., and X.B. collected the EC samples and cases. X.L. performed the data analyses. Y.W. and J.W. conducted the methodology and software parts. Y.Q., L.Z., and X.L. performed experiments. H.X., L.Z., and Y.Q. validated the results. Y.Q., Y.W., and J.W. interpreted the results. X.L., and Y.Q. wrote the original draft. J.W. reviewed and edited the paper with input from all the other authors.

## Supporting information



Supporting Information

Supplemental Table 1

Supporting Information

## Data Availability

The data that support the findings of this study are available from the corresponding author upon reasonable request.
