# Supplementary material for: Lipid‐Driven OLR1/FOXM1/FGF19 Axis Orchestrates Crosstalk in an Epithelial‐Fibroblast Positive Feedback Promoting Progesterone Resistance in Endometrial Cancer
Source: Adv Sci (Weinh). 2025 Nov 21;13(6):e11943. doi: 10.1002/advs.202511943 (PMC12866857; doi:10.1002/advs.202511943)
Supplement: Supplementary file 1 — Supporting Information [file ADVS-13-e11943-s002.pdf]

Lipid-Driven OLR1/FOXM1/FGF19 Axis Orchestrates Crosstalk in An Epithelial-Fibroblast Positive Feedback Promoting Progesterone Resistance in Endometrial Cancer

Xingchen Li[1]*, Yue Qi[1]*, Yuman Wu[1], Xinyi Bi[1], Yiqin Wang[1], Jiaqi Wang[1], Jingyuan Wang[1], Lingpu Zhang[2], Haihua Xiao[2], Jianliu Wang[1]

*Xingchen Li and Yue Qi contributed equally to this article.

Author affiliations:

[1]Department of Obstetrics and Gynecology, Peking University People's Hospital, Beijing, 100044, China

[2]Beijing National Laboratory for Molecular Sciences, State Key Laboratory of Polymer Physics and Chemistry, Institute of Chemistry Chinese Academy of Sciences, Beijing, 100190, China

Correspondence to:

Jianliu Wang

Department of Obstetrics and Gynecology, Peking University People's Hospital, No. 11, Xizhimen South Street, Xicheng District, Beijing 100044, China.

Email: wjianliu1203@163.com

Tel +86 10 8832 4474

Fax : 010-8832 4474

Supplementary figure

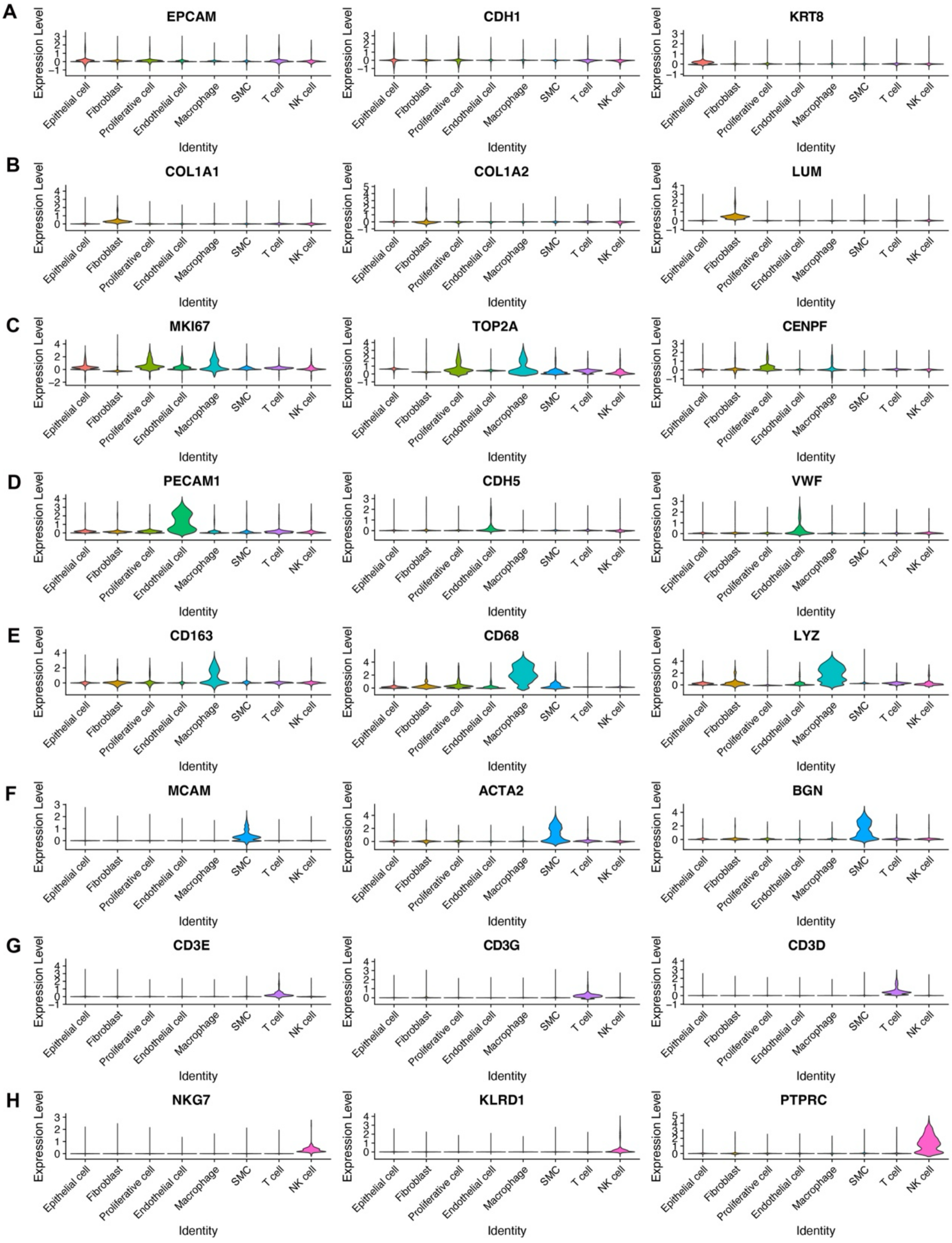

Figure S1. Violin plots showing the specific cell marker for different cell types in EC tissues. A) Epithelial cell. B) Fibroblast. C) Proliferative cell. D) Endothelial cell. E) Macrophage. F) Smooth muscle cell. G) T cell. H) NK Cell.

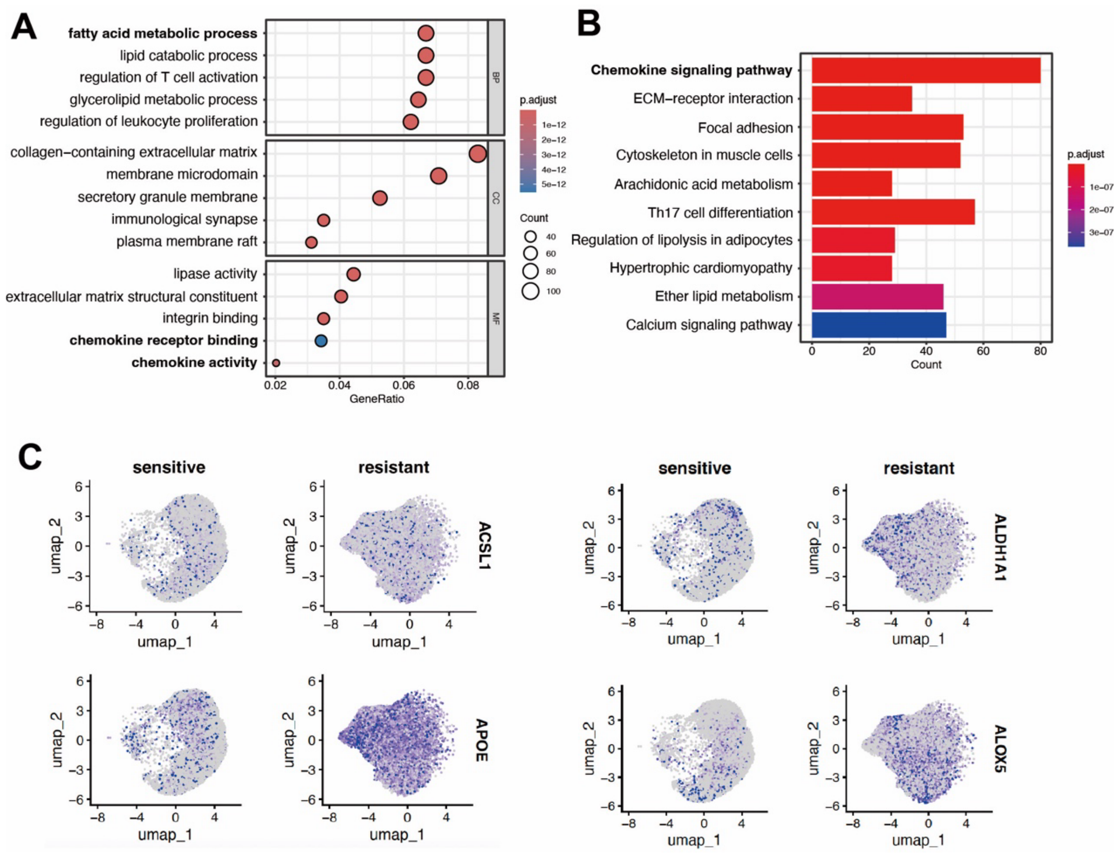

Figure S2. A-B) GO and KEGG analysis between DEGs from scRNA sequence data. C. Expression of fatty acid related genes in scRNA sequence data by umap.

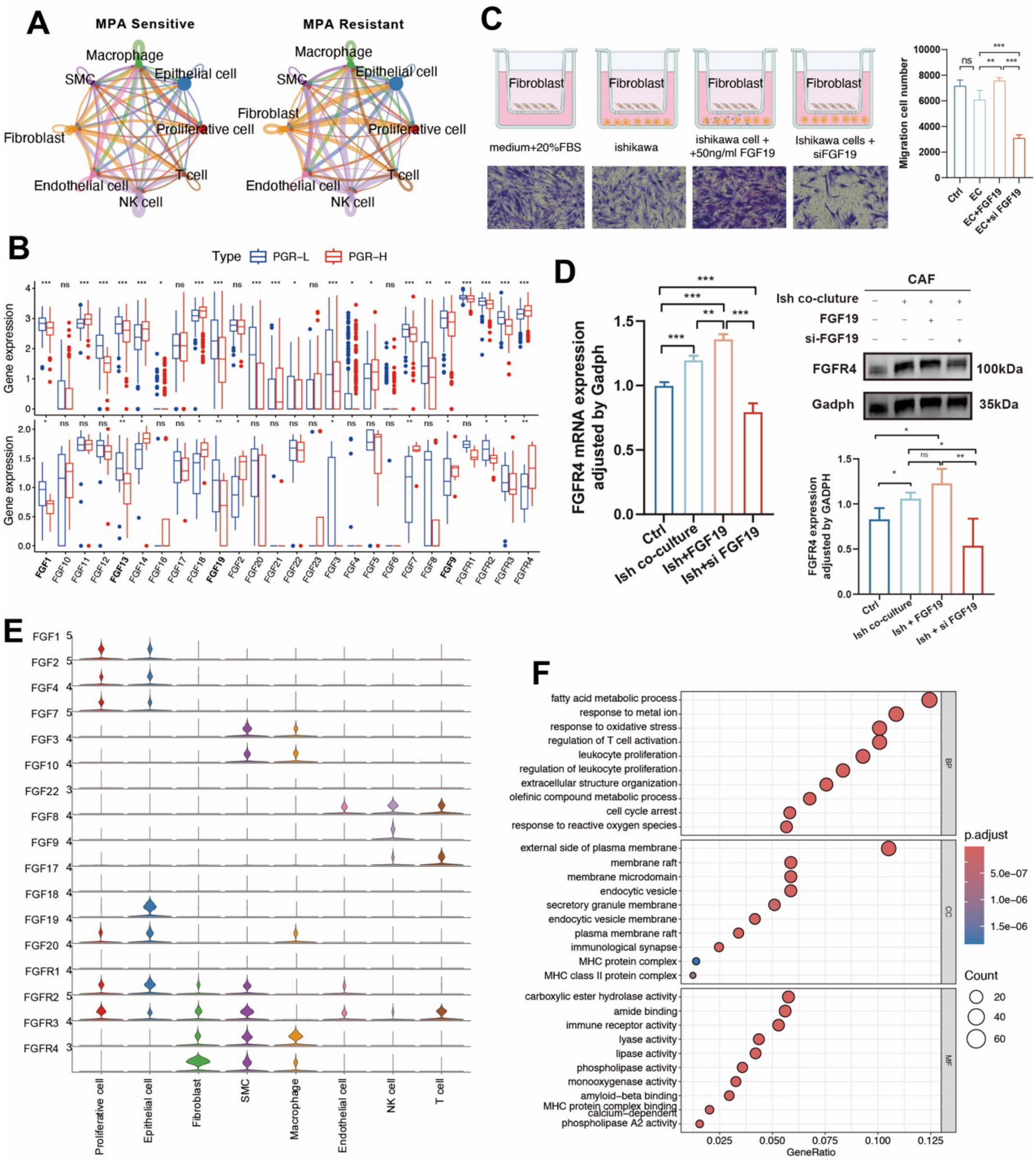

Figure S3. A) Circle plot illustrates the differential number of interactions between any two cell types in the MPA sensitive subgroup and MPA resistant group. The edges connecting the cell types are color-coded, with red indicating increased signaling in the MPA resistant compared with MPA sensitive subgroup The thickness of the lines represents the number of interactions. B) Boxplot showing the expression levels of FGF family in high and low progesterone receptor (PGR) in TCGA cohort (upper panel) and PKUPH cohort (lower panel). C) A transwell migration assay showed the migration ability influence of of cancer cell-conditioned medium (CM) and FGF19 for fibroblast. D) Expression of FGFR4 in fibroblast with or without FGF19 or conditioned mediium. E) Expression of FGF family and its receptors in scRNA data. F) GO analysis with DEGs between MPA sensitive and MPA resistant groups in fibroblast from scRNA data.

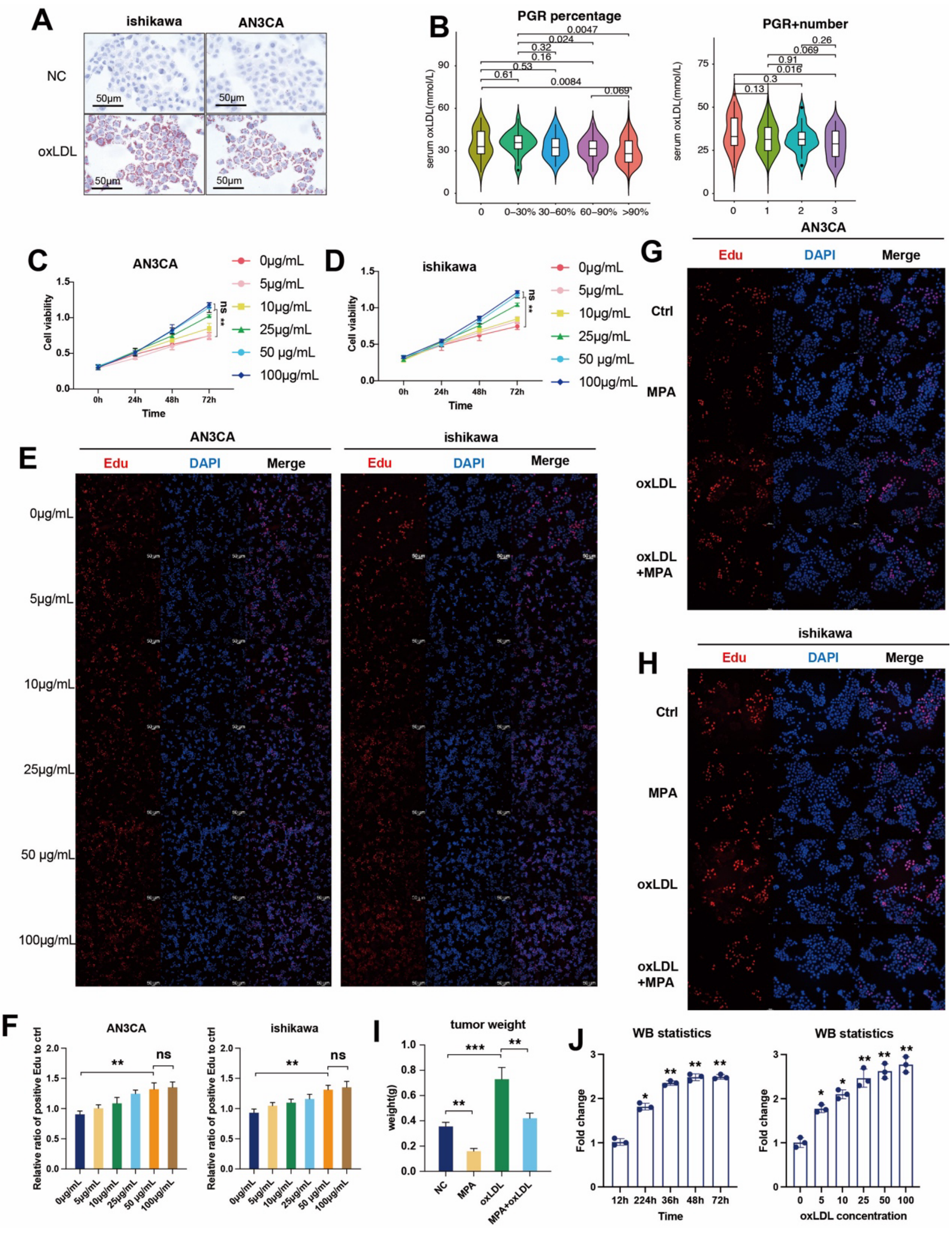

Figure S4. A) Lipid deposition evaluated by oil red in different cells treated with or without oxLDL (50μM). B) oxLDL level of patients with different expression of PR by different percentages (left panel) and plus numbers (right panel) evaluated by IHC. C-D) CCK8 assay and E-F) Edu incorporation assay evaluating different concentration of the function for oxLDL in ishikawa cell and AN3CA cell line. G-H) Edu incorporation assay of cells after the addition of MPA or oxLDL (50μM), or both treatment in ishikawa and AN3CA cell. I) Tumor weight of the corresponding group of mice in Figure 4G. J) Statistical analysis of western blot in Figure 4I.

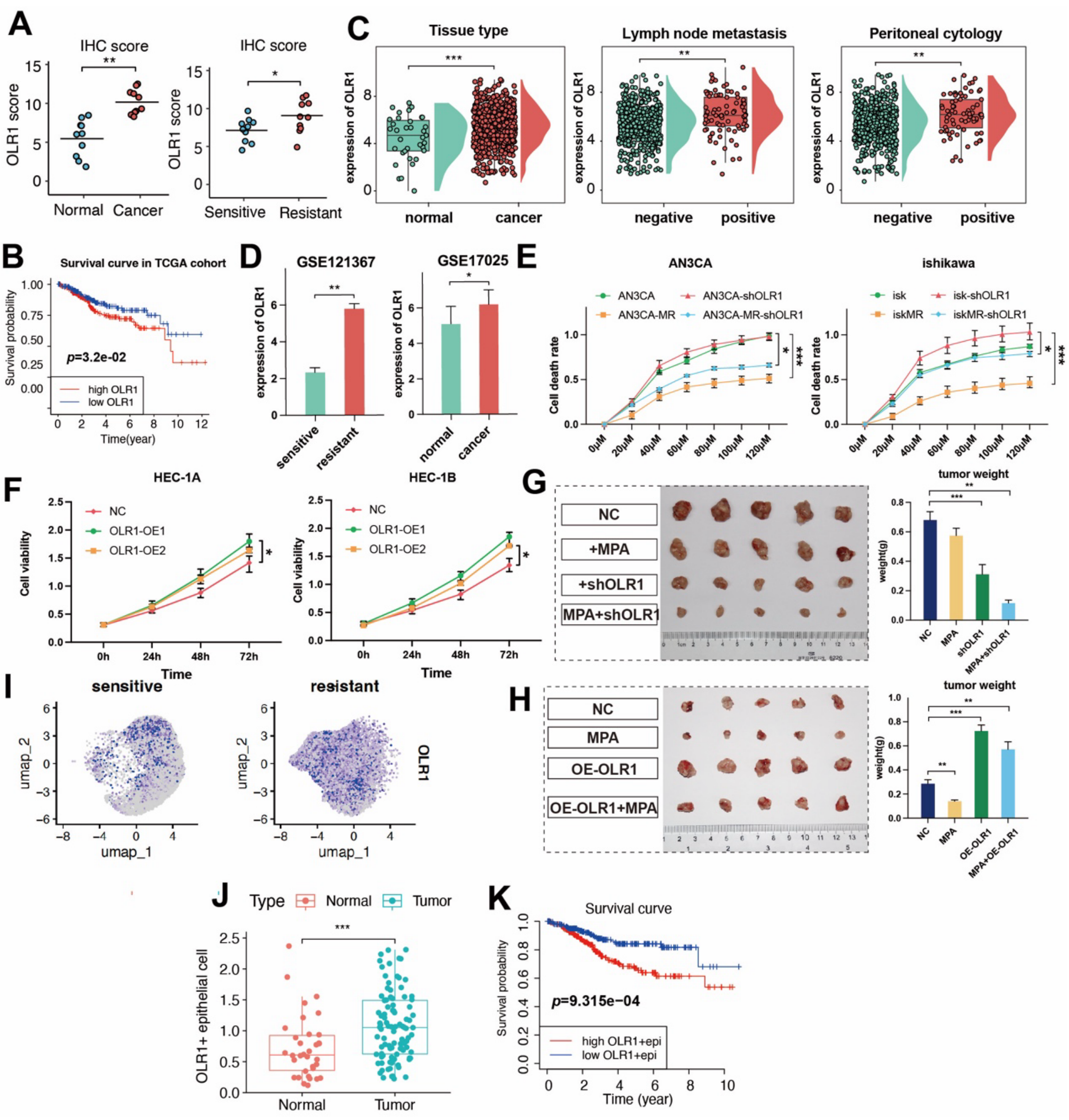

Figure S5. A) Statistical analysis of IHC score in Figure 5A. B) The Kaplan–Meier overall survival curves of TCGA patients stratified by high and low expression of OLR1. C) Expression of OLR1 in different characteristics of EC patients including different tissue types (left), lymph node metastasis (middle), and peritoneal cytology (right) in TCGA cohort. D) Expression of OLR1 in GSE121367 and GSE17025 dataset with diverse features. E）Drug resistant curve with different concentration of MPA for Ishikawa and AN3CA cells. F) Cell viability assay after OLR1 overexpression compared between control and MPA treated groups in HEC-1A (H) or HEC-1B cell lines. G-H) Representative images and tumor weight of tumor tissues isolated from mice in Figure 5. I) UMAP plots displayed the distribution of OLR1 in different sensitivity of MPA group. J) Comparison of absolute infiltration proportion of OLR1+ epithelial cells between normal (n = 35) and tumor (n = 158) in TCGA-UCEC cohort. Boxes show the median ± 1 quartile, with the whiskers extending from the hinge to the smallest or largest value within 1.5× the IQR from the box boundaries. K) The Kaplan–Meier overall survival curves of TCGA patients stratified by OLR1+ epithelial cells infiltration.

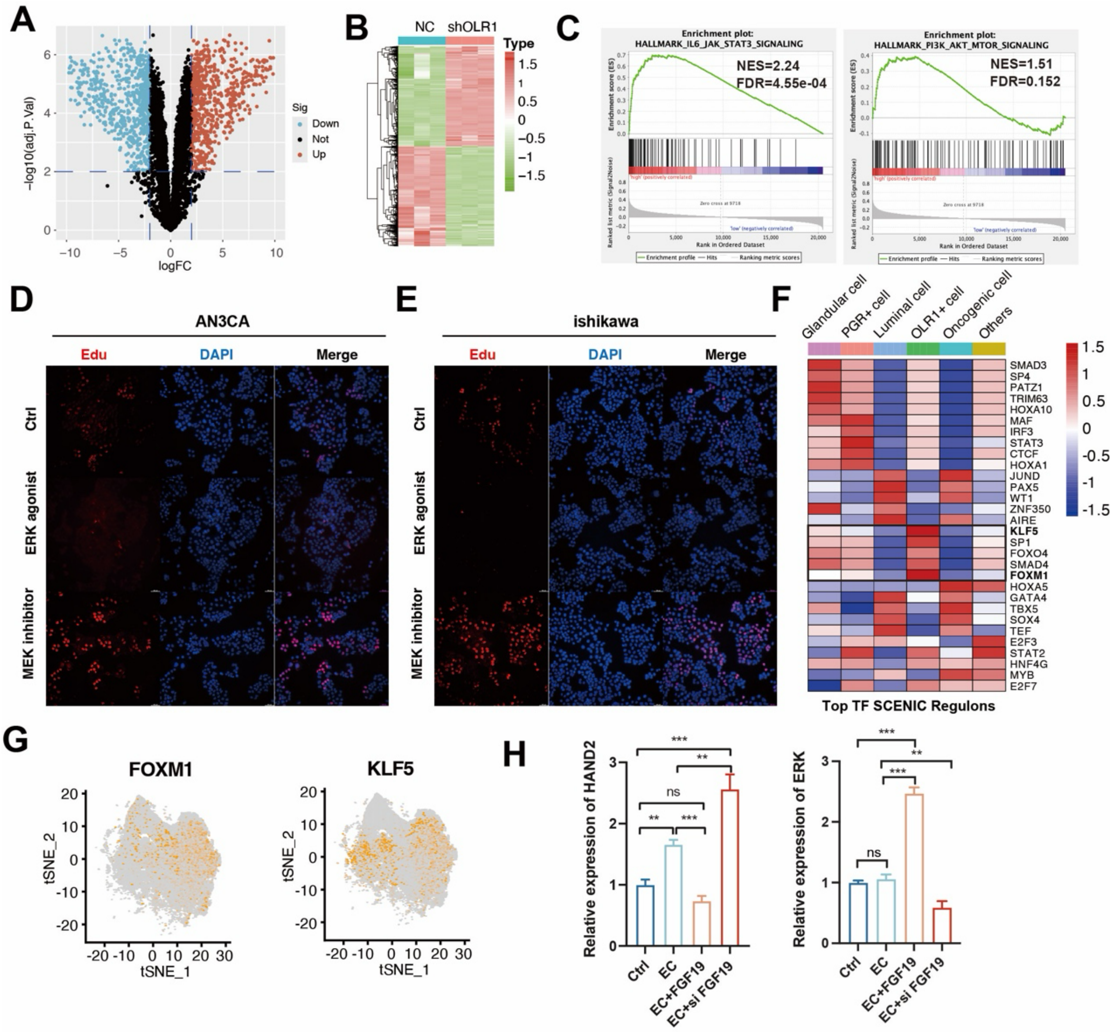

Figure S6. A) Volcano plot of RNA-seq from microarray data in Ishikawa control and shOLR1 cells. The blue dots represent up-regulated DEGs and red represent down-regulated DEGs based on a |log2FC| of ≥1. B) Hierarchically clustered heatmap of DEGs in two subgroups. C) Gene set enrichment analysis (GSEA) of groups with high and low expression of OLR1. D-E) Edu picture with MEK inhibitor or ERK agonist with two different EC cell lines. F) Heatmap shows normalized activity of top 5 TF regulons in epithelial subtype predicted by pySCENIC. TF regulons with the same as TF from venn diagram are bolded in black. G) tSNE plots showing expression of FOXM1 and KLF5-regulon. H) Relative expression of HAND2 and ERK pathway in different groups in RNA level.

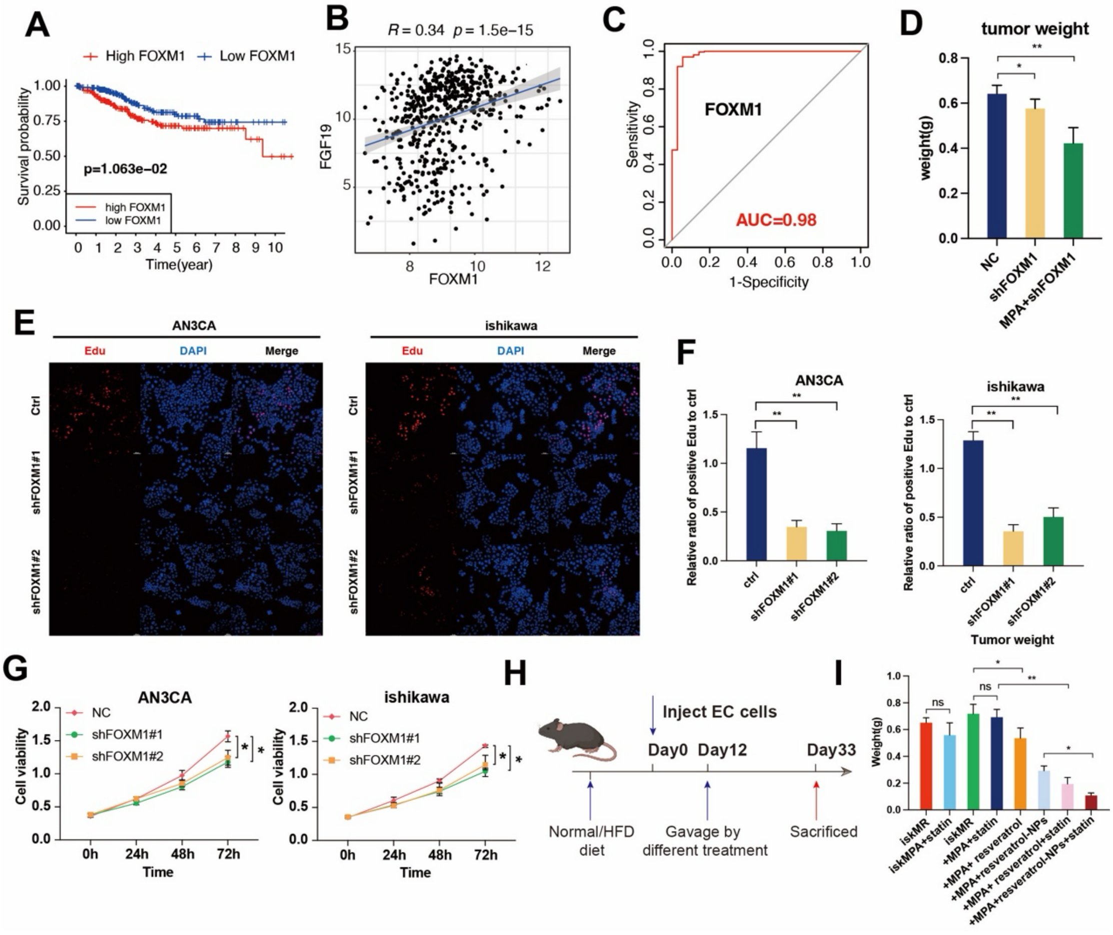

Figure S7. A) The Kaplan–Meier overall survival curves of TCGA patients stratified by high and low expression of FOXM1. B) Correlation between FOXM1 and FGF19 in TCGA cohort. C) Area under the ROC curve (AUC) evaluating the accuracy for FOXM1 predicting EC. D) Tumor weight for Figure S7C. E-F) Edu assay and statistical analysis before and after FOXM1 deletion in AN3CA or ishikawa cell lines. G) Viability assay before and after FOXM1 deletion in AN3CA and ishikawa by CCK8 experiment. H) Workflow showing the experimental process of the animal study. I) Tumor weight in different groups in Figure 7I.

Table S2 Characteristics of fertility-preservation patients

Variables	Mean+SD
Time to CR (month)	6.7 ± 6.3
Age (year)	32.6 ± 5.0
Gravida	0.5 ± 0.8
Para	0.2 ± 0.4
BMI (kg/m2)	27.2 ± 5.0
Period time (day)	25 ± 5.2
FBG (mmol/L)	5.4 ± 3.1
Insulin (mmol/L)	18.4 ± 13.2
CA125 (IU/mL)	19.1 ± 16.3
	N (%)
Status	
CR	20 (10.5%)
nonCR	170 (89.5%)
MPA resistant	
Sensitive	135 (71.1%)
Resistant	55 (28.9%)
Regularity	
Non-regular	56 (29.5%)
regular	134 (70.5%)
PCOS	
Without	124 (65.3%)
With	66 (34.7%)
Diabetes	
Without	155 (81.6%)
With	35 (18.4%)
Pathological type	
AEH	98 (51.6%)
EC G1	74 (38.9%)
EC G2	18 (9.5%)

Table S3. Primers used in qPCR

Gene	Forward primer sequence (5'-3')	Reverse primer sequence (5'-3')
GAPDH	TGACTTCAACAGCGACACCCA	CACCCTGTTGCTGTAGCCAAA
FGF19	CGGAGGAAGACTGTGCTTTCG	CTCGGATCGGTACACATTGTAG
FGF19-F1 (ChIP)	CCTGCGCATCCGTGCCGAC	GCCGCGCAGTCACGACGTC
FGF19-F2 (ChIP)	GGACGCGGGGCCCCAC	GCAGGCGGATGGGGTCG
FGF19-F3 (ChIP)	CGGACGCGGGGCCCCA	TGCGCGCAGTCACGACGC
