# Supplementary material for: Lipid‐Driven OLR1/FOXM1/FGF19 Axis Orchestrates Crosstalk in an Epithelial‐Fibroblast Positive Feedback Promoting Progesterone Resistance in Endometrial Cancer
Source: Adv Sci (Weinh). 2025 Nov 21;13(6):e11943. doi: 10.1002/advs.202511943 (PMC12866857; doi:10.1002/advs.202511943)

Figure 4I

OLR1

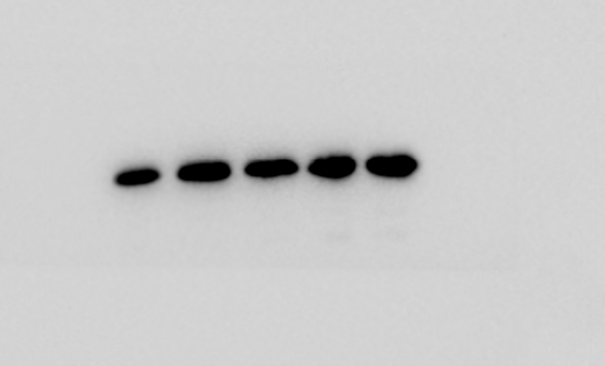

GAPDH

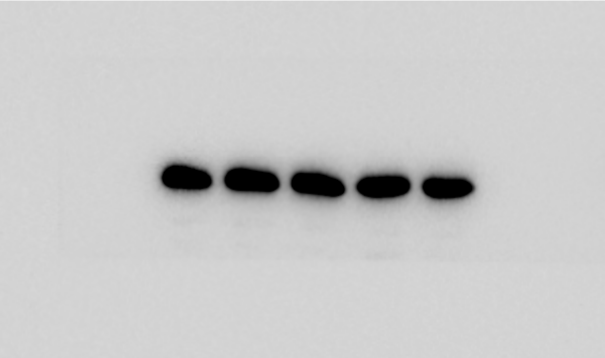

OLR1

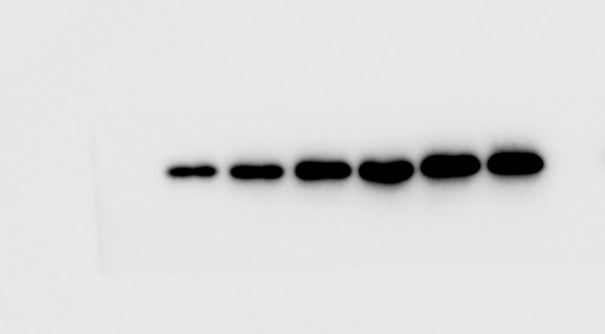

GAPDH

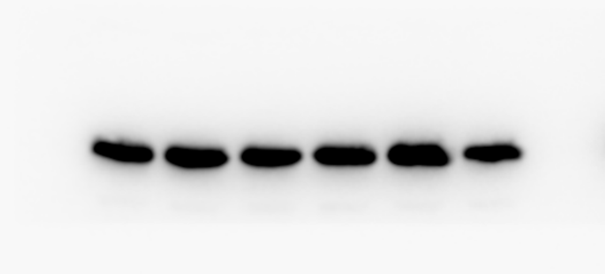

Figure6D

PI3K

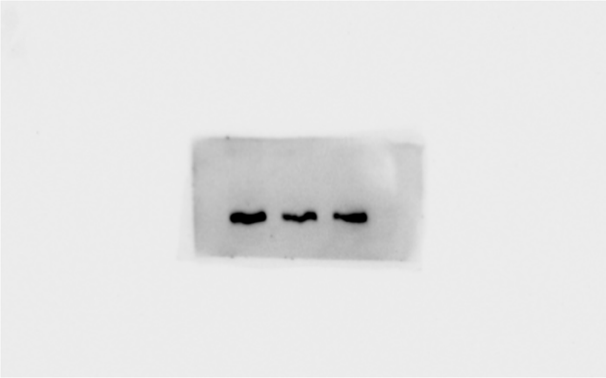

P-AKT

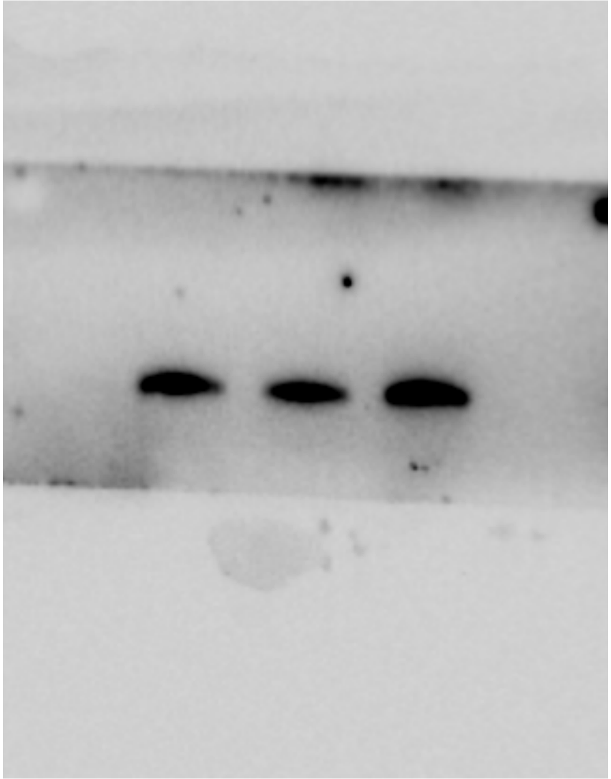

P-JAK

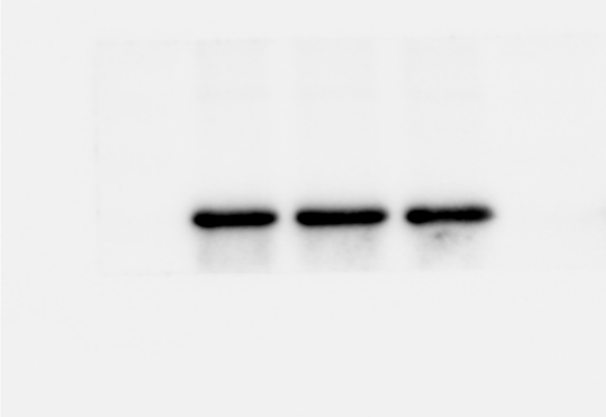

P-STAT3

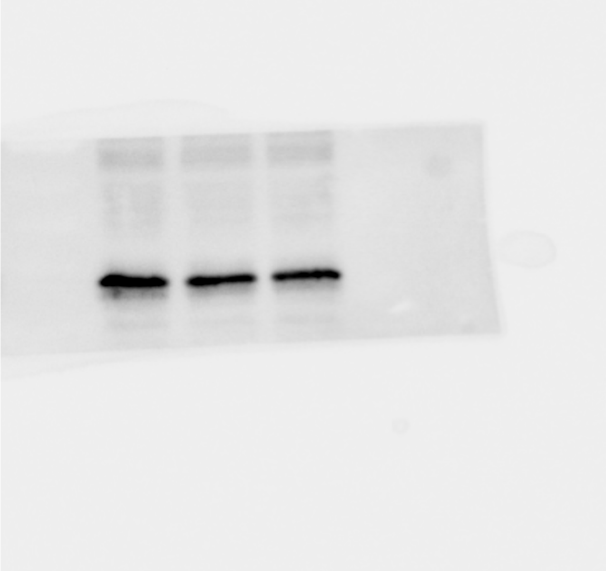

P-MEK

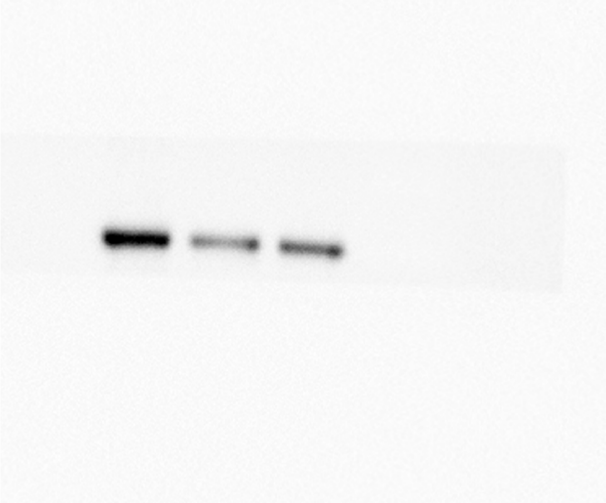

P-ERK

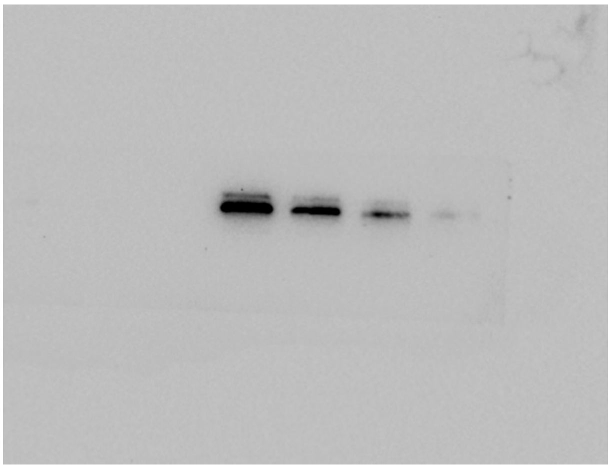

GAPDH

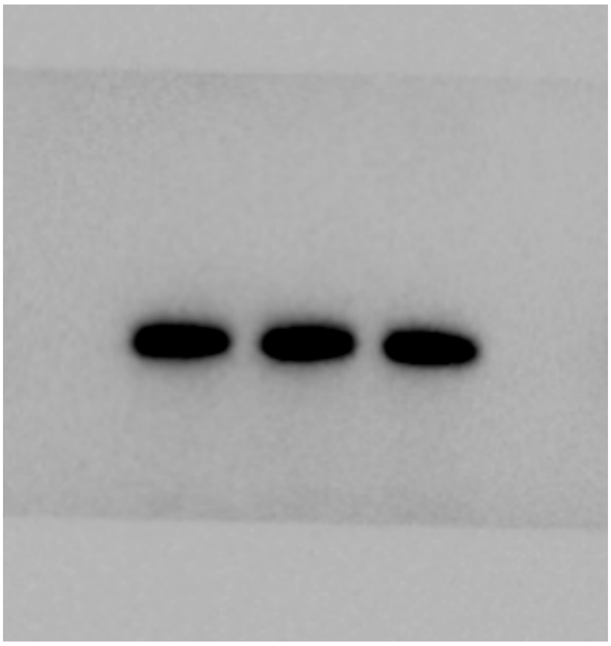

PI3K

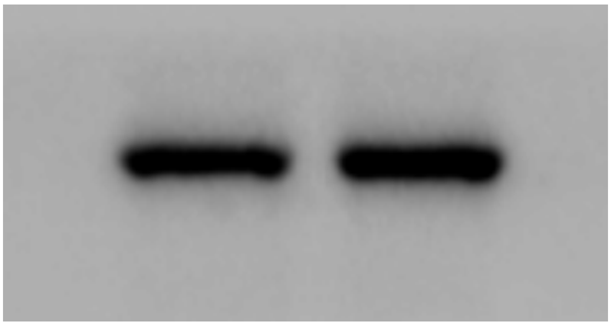

P-AKT

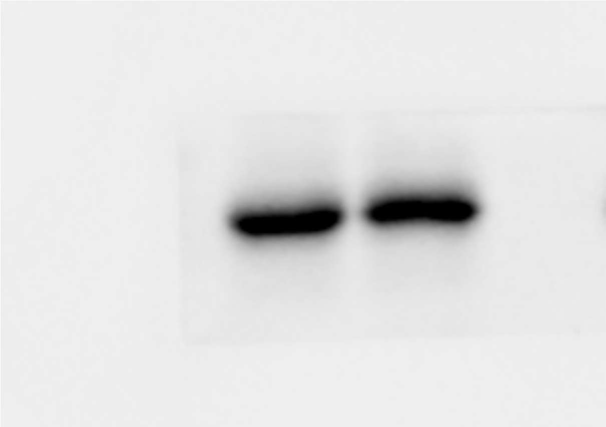

P-JAK

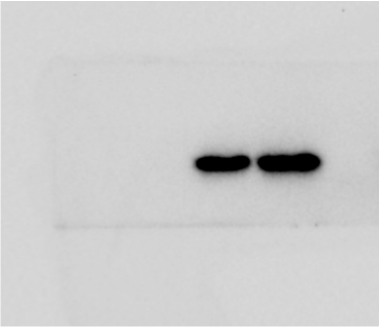

P-STAT3

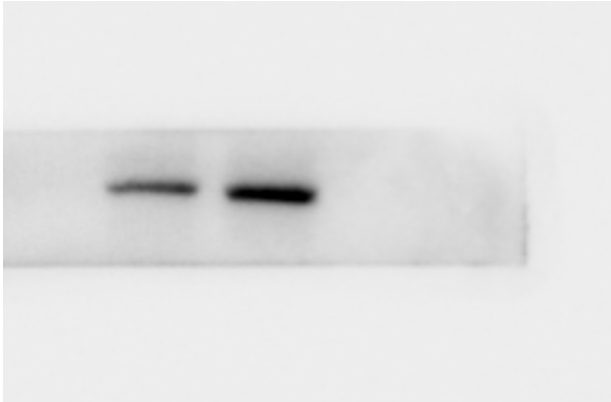

P-MEK

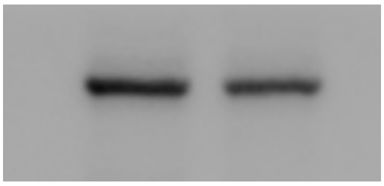

P-ERK

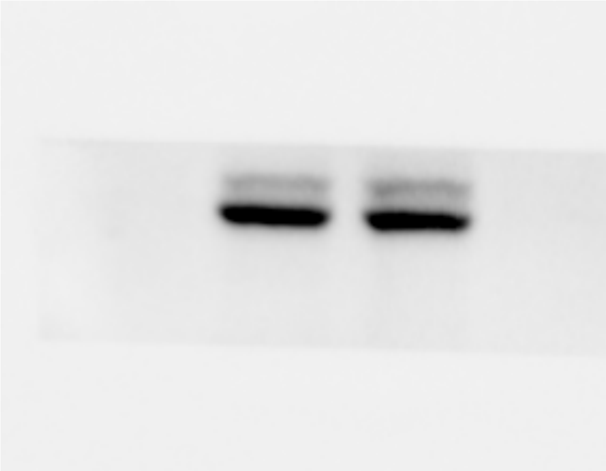

GAPDH

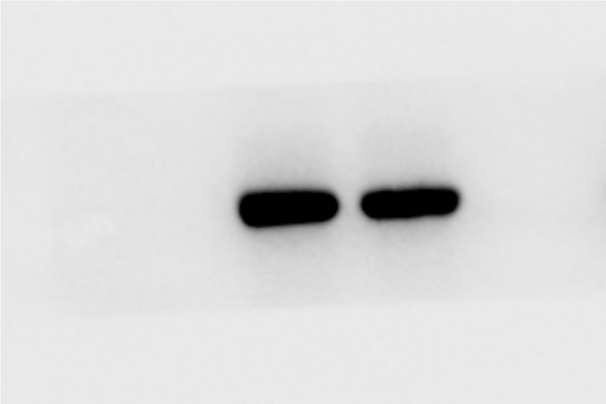

Figure 7B

FOXM1

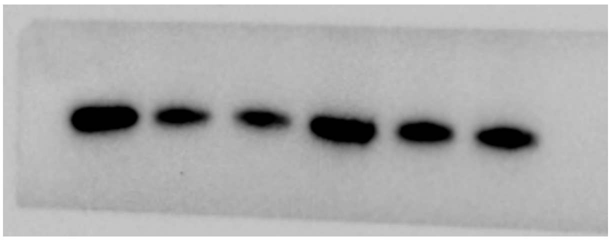

FGF19

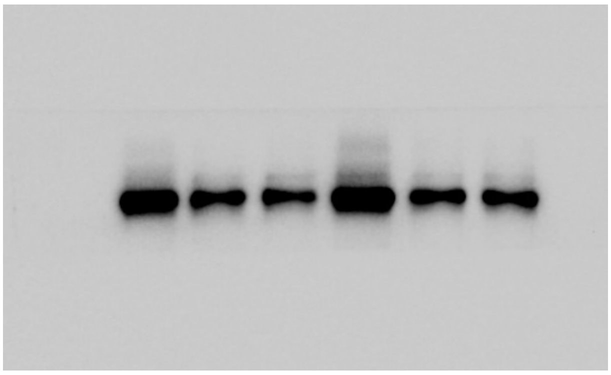

GAPDH

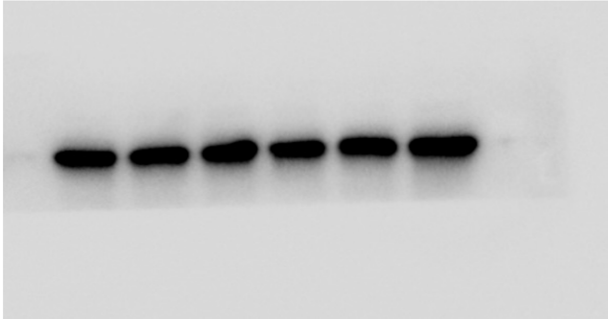

Supplement: Supplementary file 3 — Supporting Information [file ADVS-13-e11943-s001.pdf]
